# The *Arabidopsis* Lipid Transfer Protein 2 (AtLTP2) Is Involved in Cuticle-Cell Wall Interface Integrity and in Etiolated Hypocotyl Permeability

**DOI:** 10.3389/fpls.2017.00263

**Published:** 2017-02-27

**Authors:** Adélaïde Jacq, Clémentine Pernot, Yves Martinez, Frédéric Domergue, Bruno Payré, Elisabeth Jamet, Vincent Burlat, Valérie B. Pacquit

**Affiliations:** ^1^Laboratoire de Recherche en Sciences Végétales, Université de Toulouse, Centre National de la Recherche Scientifique (CNRS), Université Paul Sabatier (UPS)Castanet-Tolosan, France; ^2^Plateforme Imagerie-Microscopie, Fédération de Recherche FR3450–Agrobiosciences, Interactions et Biodiversité, Centre National de la Recherche Scientifique (CNRS), Université de Toulouse, Université Paul Sabatier (UPS)Castanet-Tolosan, France; ^3^Laboratoire de Biogenèse Membranaire, UMR 5200 CNRS Université de Bordeaux–INRA Bordeaux AquitaineVillenave d’Ornon, France; ^4^Centre de Microscopie Electronique Appliquée à la Biologie (CMEAB), Faculté de Médecine Rangueil, Toulouse III, Université Paul Sabatier (UPS)Toulouse, France

**Keywords:** *Arabidopsis thaliana*, AtLTP2, *At2g38530*, non-specific lipid transfer protein, cell wall, cuticle, plastid, plant

## Abstract

Plant non-specific lipid transfer proteins (nsLTPs) belong to a complex multigenic family implicated in diverse physiological processes. However, their function and mode of action remain unclear probably because of functional redundancy. Among the different roles proposed for nsLTPs, it has long been suggested that they could transport cuticular precursor across the cell wall during the formation of the cuticle, which constitutes the first physical barrier for plant interactions with their aerial environment. Here, we took advantage of the *Arabidopsis thaliana* etiolated hypocotyl model in which AtLTP2 was previously identified as the unique and abundant nsLTP member in the cell wall proteome, to investigate its function. At*LTP2* expression was restricted to epidermal cells of aerial organs, in agreement with the place of cuticle deposition. Furthermore, transient AtLTP2-TagRFP over-expression in *Nicotiana benthamiana* leaf epidermal cells resulted in its localization to the cell wall, as expected, but surprisingly also to the plastids, indicating an original dual trafficking for a nsLTP. Remarkably, in etiolated hypocotyls, the *atltp2-1* mutant displayed modifications in cuticle permeability together with a disorganized ultra-structure at the cuticle-cell wall interface completely recovered in complemented lines, whereas only slight differences in cuticular composition were observed. Thus, AtLTP2 may not play the historical purported nsLTP shuttling role across the cell wall, but we rather hypothesize that AtLTP2 could play a major structural role by maintaining the integrity of the adhesion between the mainly hydrophobic cuticle and the hydrophilic underlying cell wall. Altogether, these results gave new insights into nsLTP functions.

## Introduction

In many instances, lipid synthesis and metabolism require the cooperation of various organelles and cellular structures implicating the transport of lipidic precursors/intermediates from one cellular compartment to another (Ohlrogge and Browse, 1995). Lipid Transfer Proteins (LTPs), primarily defined by their capability of transferring lipids between lipid bilayers *in vitro*, are good candidates to convey hydrophobic lipids within the hydrophilic intracellular spaces (Kader, 1996). In addition, plant non-specific LTPs (nsLTPs) share highly conserved structures characterized by four disulfide bridges formed by eight cysteine residues that delineate an internal hydrophobic cavity able to bind various hydrophobic compounds *in vitro* (Carvalho and Gomes, 2007; Liu et al., 2015). The nsLTPs are encoded by a large multigenic family in flowering plants (Boutrot et al., 2008; Edstam et al., 2011; Liu et al., 2015). Based on (i) sequence similarity, (ii) calculated molecular mass of the mature protein, (iii) presence or not of a glycosylphosphatidylinositol (GPI) modification site, and (iv) intron position, terrestrial plant nsLTPs have been grouped in 10 clades (Edstam et al., 2011). Most plant nsLTPs possess a predicted N-terminal signal peptide to target them to the secretory pathway (Edstam et al., 2011). The absence of identification of additional sorting signals to endomembrane compartments has suggested their bulk transport to the extracellular compartment and particularly to the cell wall. Some nsLTPs have been detected to the outer side of the plasma membrane where they are attached by a GPI-anchor (Liu et al., 2015). However, a recent study has shown that a sunflower nsLTP, could be re-localized intra-cellularly by endocytosis during seed imbibition, thus possibly contributing to intra-cellular transport of lipids (Pagnussat et al., 2012).

The knowledge of nsLTPs has considerably increased in recent years with the first characterization of a NMR 3D-structure, insight into their biochemical properties, description of spatio-temporal patterns of expression, and sub-cellular localization (Yeats and Rose, 2008; Liu et al., 2015). Several roles have been proposed for a few nsLTPs in diverse physiological processes, for example, cell wall loosening (Nieuwland et al., 2005), pollen exine assembly (Huang et al., 2013), sexual reproduction (Chae et al., 2010), seed development (Wang et al., 2015), response to biotic/abiotic stress (Lee et al., 2009; Guo et al., 2013; Deeken et al., 2016), symbiotic nodulation (Lei et al., 2014), and cutin and wax assembly. In the latter case, a few studies provided experimental evidence of a possible role for two plasma membrane-localized GPI-anchored nsLTPs (AtLTPG/LTPG1, AtLTPG2) in the control of the cuticle lipid composition (Debono et al., 2009; Lee et al., 2009; Kim et al., 2012). However, the description of nsLTPs mode of action remains unclear and the identification of their genuine physiological substrates is still lacking. The difficulties encountered to decipher their function are probably due to the large size of the nsLTP multigenic family implicating possible functional redundancy.

The cuticle provides a primary barrier to prevent non-stomatal water-loss, but also plays various roles during development and in response to environmental stresses (Yeats and Rose, 2013). This protective layer is a complex structure mainly composed of a lipid polymer, i.e., cutin, which is impregnated and covered by cuticular waxes (Yeats and Rose, 2013; Lee and Suh, 2015; Fernandez et al., 2016). The cuticle coats the outermost layer of periclinal cell walls of all aerial organ epidermal cells. The biosynthetic pathways of the fatty acid derivatives constituting the waxes and the cutin polymer are now well described (Yeats and Rose, 2013), but their intra-cellular trafficking is still poorly understood. In addition, if the transport across the plasma membrane of both wax and cutin precursors has been shown to be dependent on ATP-binding cassette (ABC) transporters, the transfer through the cell wall to the cuticle and the assembly mechanisms within the cuticle are still a matter of debate (Samuels et al., 2008; Bernard and Joubes, 2013; Domínguez et al., 2015). One particular paradigm that remains to be solved is how the hydrophobic cuticle layer is structurally stabilized next to the highly hydrophilic polysaccharidic cell wall.

Etiolated hypocotyls of *Arabidopsis thaliana* constitute a well-documented model of cell elongation (Gendreau et al., 1997; Refregier, 2004; Derbyshire et al., 2007; Pelletier et al., 2010). During a short period of active growth in darkness, no cell division occurs in hypocotyls and, consequently, growth only proceeds by cell elongation. Furthermore, the outer cuticle-periclinal wall zone has been observed to be thicker than in light-growth condition (Gendreau et al., 1997). Thus, in addition to their primary interest for elongation studies, etiolated hypocotyls could be a new attractive model to gain insight into the understanding of cuticle formation.

In this study, we have taken advantage of the fact that AtLTP2 (NP_181387/At2g38530) is the unique and abundant nsLTP that has been identified in the cell wall proteome of etiolated hypocotyls (Irshad et al., 2008) among the 49 (Boutrot et al., 2008) or 79 (Edstam et al., 2011) nsLTPs of *A. thaliana*. We have assumed that this feature would facilitate the search for its function and the exploration of its possible role in cuticle biogenesis. We provide evidence for spatio-temporal restricted accumulation of *AtLTP2* transcripts in epidermal cells where the cuticle is synthesized and for a dual sub-cellular localization of AtLTP2 to the cell wall and plastids. We also use a reverse genetics approach to demonstrate its contribution to the control of the permeability of etiolated hypocotyls, but not to the cuticle composition and content. Finally, a topochemical ultra-structural study enables us to propose a role of AtLTP2 in maintaining the integrity of the cuticle-cell wall interface.

## Results

### Generation of an *atltp2-*1 Mutant Line Strongly Affected at the Transcript and Protein Levels

**FIGURE 1:**
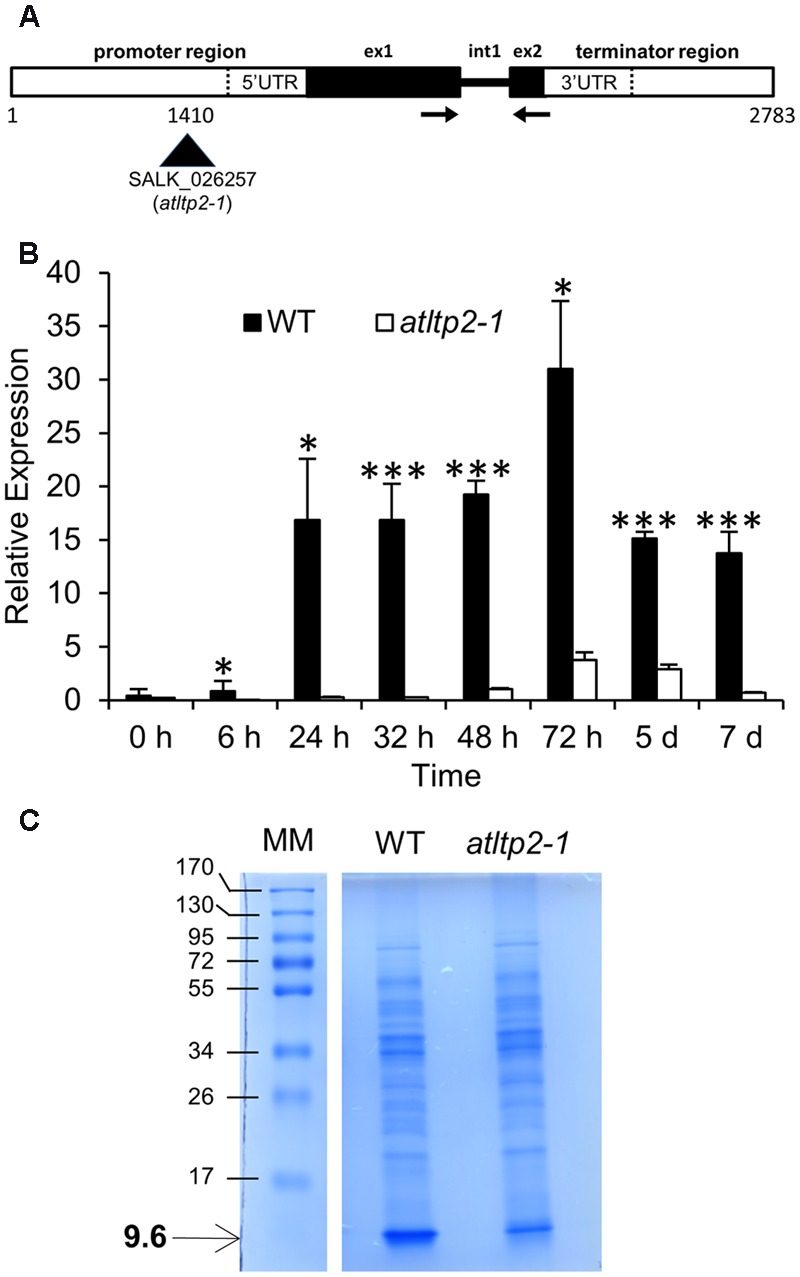
***atltp2-1* is a strong knock-down mutant. (A)** Organization of the *AtLTP2* (*At2g38530*) genomic sequence showing the T-DNA insertion in the SALK_026257 (*atltp2-1*) mutant. The displayed 2,783 bp sequence containing native regulatory sequences was cloned and used for complementation of *atltp2-1*. Legend: exon (ex); intron (int); UTR (untranslated region). The arrows show the position of primers used for RT-qPCR. **(B)** Relative *AtLTP2* expression was determined by RT-qPCR for etiolated seedling kinetics at eight developmental stages in wild type (WT) (black) and *atltp2-1* (white). Results represent the mean values ± SD (*n* = 3). Significant differences were assessed by a Student’s *t*-test (^∗^*p*-value < 0.05; ^∗∗∗^*p*-value < 0.001). **(C)** 1D-electrophoresis (1D-E) profiles of proteins extracted from cell walls of 5-day-old WT and *atltp1-2* seedlings. The numbers on the left indicate the position of protein molecular mass markers (MM) in kDa. The arrow shows the position of the 9.6 kDa band containing AtLTP2.

In order to investigate the function of *AtLTP2*, the *A. thaliana* SALK_026257 insertion line in which the *AtLTP2* promoter is disrupted by a T-DNA insertion (**Figure 1A**), was studied. A homozygous mutant line (*atltp2-1*) and its wild type counterpart (WT) were selected during a genotyping screen. The genomic sequence of *AtLTP2 (*2,783 bp), including native regulatory sequences (promoter and terminator sequences) and the unique intron (**Figure 1A**), was used to transform homozygous *atltp2-1* plants, finally leading to the selection of 12 homozygous independent complemented lines (comp). Three of them (i.e., 5′2, 6′4, and 14′3) were selected for further studies.

Firstly, *AtLTP2* expression was investigated by RT-qPCR on etiolated seedlings during 7 days, timing starting at the time of transfer to dark after synchronization of germination by light exposure for 4 h. In WT seedlings, the level of accumulation of *AtLTP2* transcripts showed a significant 15-fold increase between 24 and 48 h, and peaked at 72 h before decreasing to the same level as at 48 h (**Figure 1B**). A 5- to 64-fold reduction in the accumulation of *AtLTP2* transcripts was observed in *atltp2-1* seedlings as compared to WT between 24 h and 7 days (**Figure 1B**). An *AtLTP2* expression pattern similar to that of WT was retrieved in the three selected independent complemented lines (Supplementary Figure S1).

To further characterize these lines, we have performed a MALDI-TOF MS analysis of proteins extracted from cell walls of 5-day-old etiolated seedlings of WT and *atltp2-1*. Proteins were separated by 1D-electrophoresis (1D-E). A band around 9.6 kDa, in which AtLTP2 was previously identified (Irshad et al., 2008), was clearly visible after Coomassie blue staining in the WT extract, but appeared much fainter in the *atltp2-1* extract (**Figure 1C**). MALDI-TOF MS analysis after tryptic digestion of the 9.6 kDa band from WT displayed four peptides specific for AtLTP2 allowing its identification: one major peptide (m/z 955.5321) and three minor ones (m/z 1008.4359, 992.4441, and 1236.5724; Supplementary Figure S2). In *atltp2-1*, only a small peak corresponding to the major peptide (m/z 955.5321) was detected in the 9.6 kDa band, suggesting that this band also contained AtLTP2. No other protein could be identified in the 9.6 kDa band. Even though MALDI-TOF MS was not quantitative, combined SDS-PAGE and MALDI TOF MS analyses indicated that the amount of AtLTP2 was lower in *atltp2-1* in comparison to WT.

Altogether, these experiments have confirmed the strong reduction of the level of accumulation of *AtLTP2* gene products in *atltp2-1*, both at the transcript and protein levels. Furthermore, these results have demonstrated that the selected *AtLTP2* genomic region, carrying the native regulatory sequences, was sufficient to rescue the WT *AtLTP2* expression level in *atltp2-1*, and that the use of these genetic lines (WT, *atltp2-1*, and comp) was relevant for the following phenotyping studies.

### In Dark-Grown Seedlings, *AtLTP2* Expression Is Restricted to the Epidermal Cells of Aerial Organs

**FIGURE 2:**
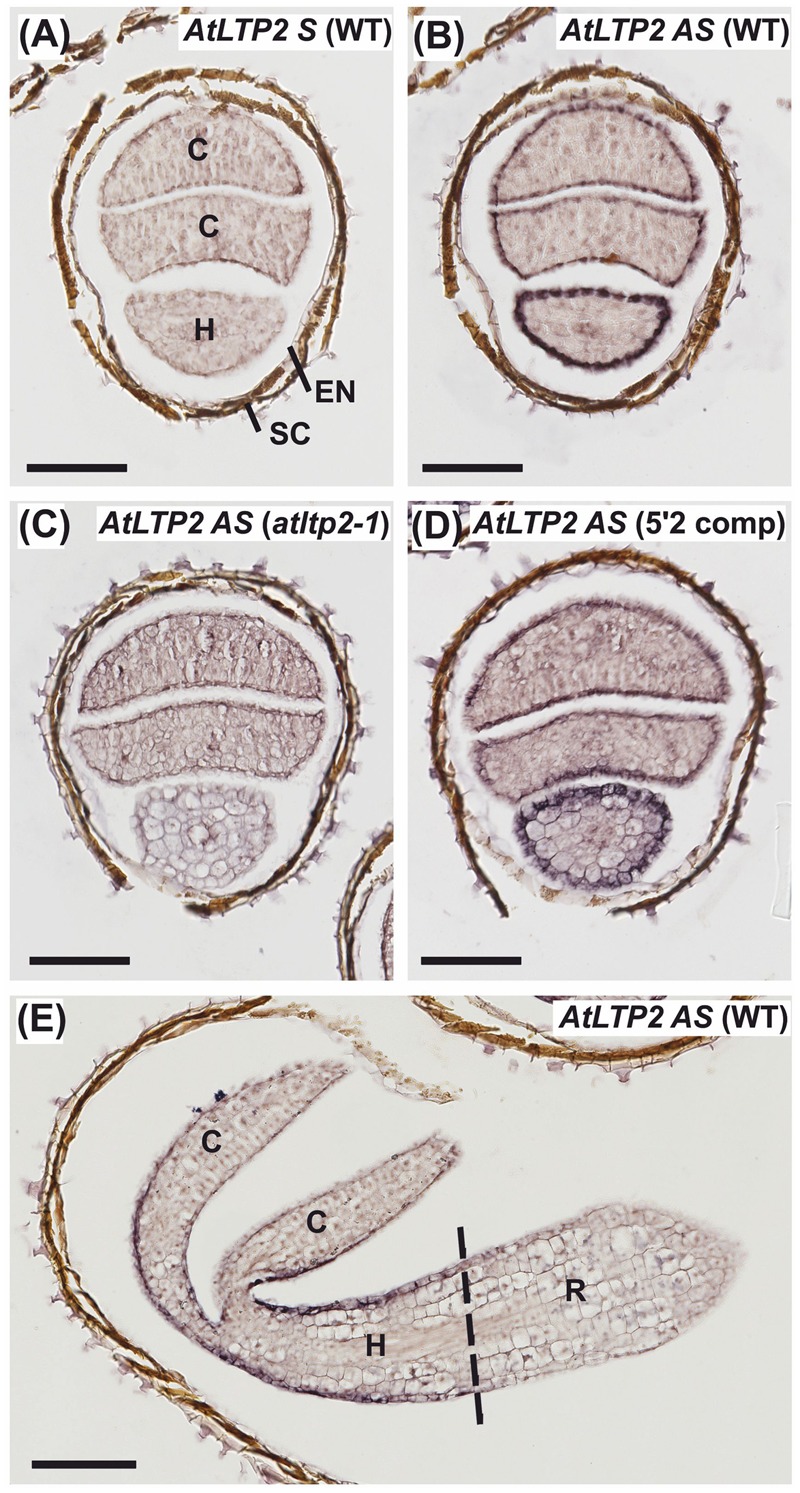
***AtLTP2* expression is restricted to epidermal cells of cuticle-containing hypocotyl and cotyledons from etiolated seedlings. (A–E)** ISH was performed with *AtLTP2* antisense (*AS*) and sense (*S*) probes on 24-h-old serial cross-sections **(A–D)** and 30-h-old etiolated seedling longitudinal sections **(E)** of WT, *atltp2-1* and one complemented line (5′2 comp). The *AtLTP2 S* probe **(A)** allowed showing the low hybridization background level within the etiolated seedling tissues (C, cotyledons; H, hypocotyls; R, radicle) and in the endosperm (EN), and to visualize the natural brown color of the seed coat (SC). Scale bars: 100 μm.

To characterize the spatio-temporal expression pattern of *AtLTP2, in situ* RNA hybridization (ISH) was performed on serial sections from young etiolated seedlings (24 or 30-h-old, timing starting as described for RT-qPCR experiments). The *AtLTP2* sense (S) probe, used as a negative control, displayed no significant background on WT seedling cross-sections (**Figure 2A**). The natural brown color observed on seed coats in all samples did not correspond to ISH labeling (**Figure 2**). By comparison, the subsequent serial section hybridized with the *AtLTP2* antisense (AS) probe displayed an intense purple signal restricted to the epidermis of both the hypocotyl and, to a lesser extent, the cotyledons (**Figure 2B**). Cross-section of the *atltp2-1* seedlings, at the same developmental stage, did not show such a labeling with the *AtLTP2* AS probe (**Figure 2C**), in agreement with the RT-qPCR data (**Figure 1B**). The epidermal *AtLTP2* hybridization signal was retrieved in the epidermis from aerial organs of the 5′2 complemented line (**Figure 2D**). Interestingly, the ISH performed with the *AtLTP2* AS probe on a longitudinal section of WT etiolated seedlings clearly illustrated the restriction of the *AtLTP2* expression to epidermal cell of aerial organs which are covered by a cuticle (hypocotyl and cotyledons) and the absence of such an expression in the cuticle-deprived radicle (**Figure 2E**).

### AtLTP2 Is Dually Localized to the Cell Wall and the Plastids

**FIGURE 3:**
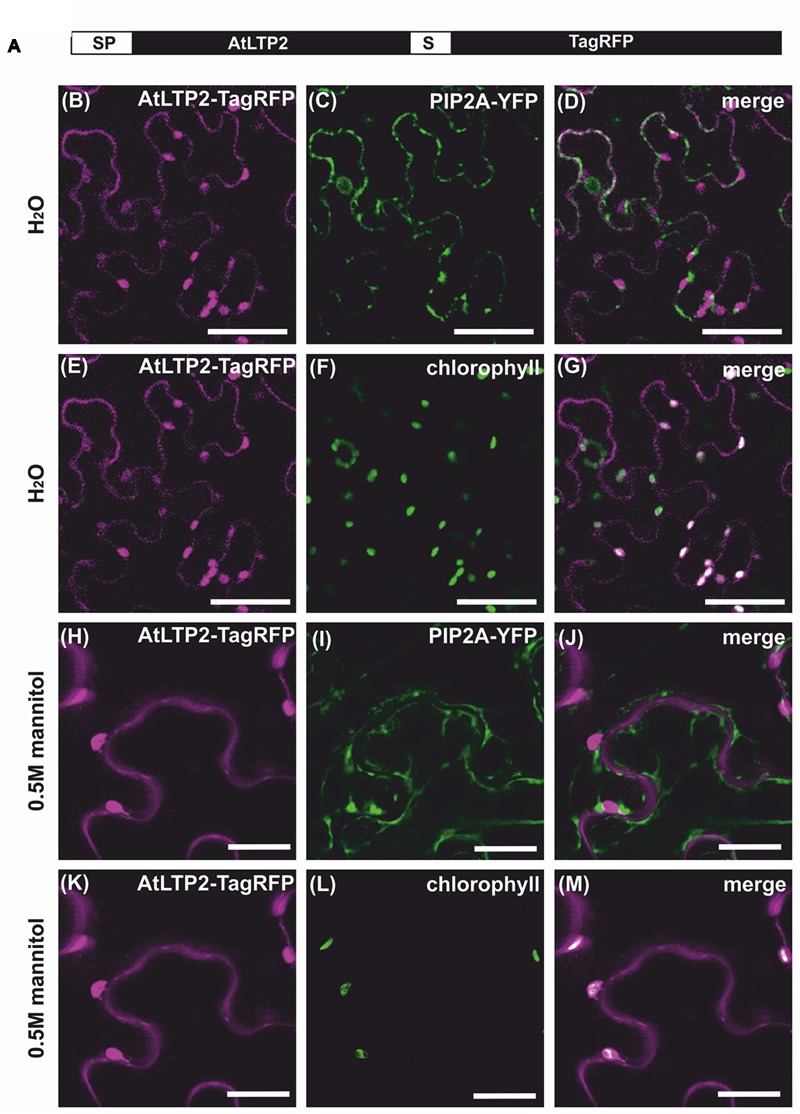
**AtLTP2-TagRFP is dually targeted to the cell wall and the plastids.** The fusion protein AtLTP2-TagRFP displayed in **(A)** was produced together with the plasma membrane (PM) marker PIP2A-YFP in *Nicotiana benthamiana* leaves. Detached leaves were observed under hypotonic **(B–G)** or hypertonic **(H–M)** conditions. The Tag-RFP fluorescence signal is false-colored in magenta in the left column. The PM and the plastid markers (chlorophyll auto-fluorescence) are false-colored in green in the second column and the merge is presented in white in the right column. Note that under plasmolysis, the AtLTP2-TagRFP fluorescence in the cell wall is excluded from the PM fluorescence of PIP2A-YFP and that the additional co-localization of AtLTP2-TagRFP fluorescence with the chlorophyll auto-fluorescence indicates its plastid localization. Scale bars: 50 μm.

Like most nsLTPs, AtLTP2 carries a predicted N-terminal signal peptide of 23 amino acids in length most likely directing AtLTP2 to the secretory pathway (Edstam et al., 2011). The mature 9.6 kDa protein accumulated in the cell wall proteome of dark-grown hypocotyls (Irshad et al., 2008) in agreement with the assumed localization of nsLTPs (Kader, 1996). In order to confirm its sub-cellular localization *in vivo*, AtLTP2 fused to the fluorescent protein TagRFP (Red Fluorescent protein, Merzlyak et al., 2007) was transiently produced in *Nicotiana benthamiana* leaves (**Figure 3A**). The TagRFP is characterized by its low 3.8 pKa especially well-adapted to fluorescence emission in various cell compartments including at the acidic pH of the cell wall compartment (Albenne et al., 2014).

A specific TagRFP fluorescence signal was observed at the cell periphery, but was difficult to distinguish from the co-transformed PIP2A-YFP plasma membrane marker (Nelson et al., 2007) (**Figures 3B–D**). To overcome this difficulty, the detached transformed leaves were plasmolysed in 0.5 M mannitol, allowing the distinction between the peripheral fluorescence of the AtLTP2-TagRFP fusion protein and the retracted PIP2A-YFP fluorescence (**Figures 3H–J**), thus clearly demonstrating the cell wall localization of AtLTP2-TagRFP. Surprisingly, we also observed in the same cells a strong additional fluorescent signal of AtLTP2-TagRFP that co-localized with the chlorophyll auto-fluorescence both in hypotonic and hypertonic conditions (**Figures 3E–G,K–M**). In addition, *AtLTP2* fused to the YFP coding sequence was transiently expressed in *N. benthamiana* leaves (Supplementary Figure S3). AtLTP2-YFP was strongly visualized in plastids, thus confirming this unexpected localization (Supplementary Figure S3). However, certainly due to the YFP instability in the cell wall acidic compartment (pKa 6.9; Albenne et al., 2014), AtLTP2-YFP fluorescence was not detected in cell wall contrary to that of AtLTP2-TagRFP. Altogether, these results showed that AtLTP2 had an original dual localization to the cell wall and the plastids.

**FIGURE 4:**
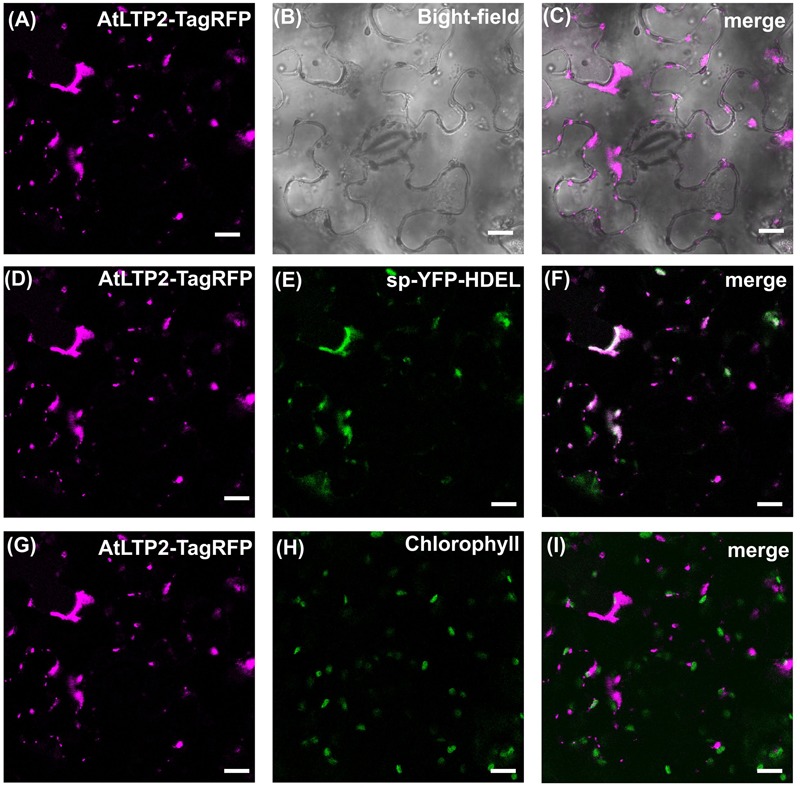
**The secretory pathway is involved in both cell wall and plastids *At*LTP2-TagRFP localization.** The AtLTP2-TagRFP fusion protein was produced together with the endoplasmic reticulum (ER) marker sp-YFP-HDEL in *N. benthamiana* leaves which were subjected to Brefeldin A (BFA) treatment. The AtLTP2-TagRFP fluorescence signal is false-colored in magenta in **(A,D,G)**. The bright-field **(B)** is displayed to visualize the cell morphology. ER marker **(E)** and chlorophyll auto-fluorescence **(H)** are false-colored in green and the merge is presented in white in the right column **(C,F,I)**. Note that in presence of BFA, AtLTP2-TagRFP fluorescence is restricted to the ER-Golgi hybrid and BFA compartments. Scale bars: 40 μm.

To further clarify the mechanism of AtLTP2 transport to the plastids, AtLTP2-TagRFP was produced in *N. benthamiana* leaves in the presence of Brefeldin A (BFA), a drug used to prevent the endoplasmic reticulum (ER)-to-Golgi trafficking (Daskalova et al., 2010). In presence of BFA (**Figure 4**), the AtLTP2-TagRFP fluorescence was strictly restricted to the ER-Golgi hybrid and BFA compartments (Nebenführ et al., 2002; Ritzenthaler et al., 2002). It co-localized with the sp-YFP-HDEL marker of the ER compartment (Nelson et al., 2007) suggesting that AtLTP2-TagRFP was redistributed in the oversized ER-Golgi modified structures as previously described (Ritzenthaler et al., 2002). In the control experiment without BFA, AtLTP2-TagRFP was still observed in the cell wall and in plastids (Supplementary Figure S4). In presence of BFA, AtLTP2-TagRFP was no longer localized to the cell wall and to plastids suggesting that AtLTP2 needed to go through the ER/Golgi apparatus to reach these two compartments. Therefore, it was assumed that the full length AtLTP2 pre-protein was first targeted to the secretory pathway thanks to its signal peptide, prior to be sorted to both the cell wall and plastids.

### Reduced Expression of *AtLTP2* Leads to an Increase in Cuticle Permeability without Major Modification of the Cuticle Lipid Profile

**FIGURE 5:**
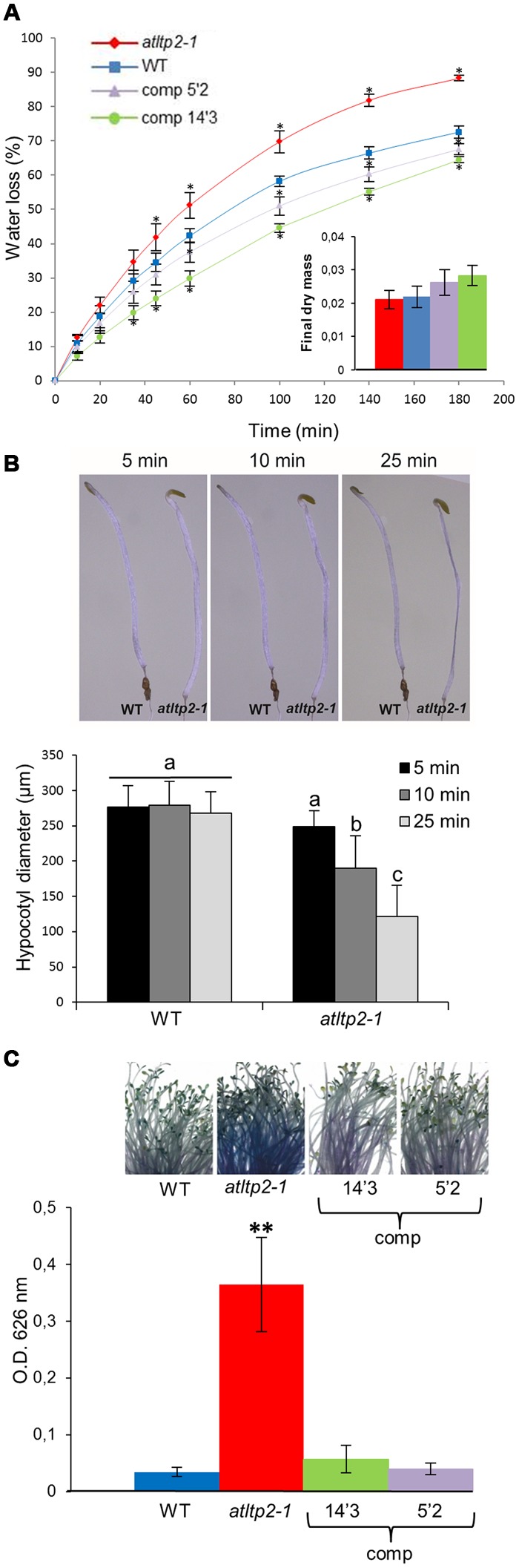
***atltp2-1* etiolated seedlings show increased water-loss and toluidine blue (TB) permeability. (A)** Water-loss kinetics of WT, *atltp2-1* and complemented lines (5′2 and 14′3; expressed as % of fresh mass) were performed at 25°C. Points represent mean values ± SE (*n* = 3). Significant differences from WT were assessed by a Wilcoxon rank-sum test (^∗^*p*-value ≤ 0.05). Dry mass was measured after 3 days at 25°C. No significant differences between the different lines were assessed using a Wilcoxon rank-sum test (*p*-value > 0.05) **(B)** *Arabidopsis thaliana* etiolated seedlings from 5-day-old WT and *atltp2-1* were imaged along a 25 min kinetics following exit from the *in vitro* conditions. *atltp2-1* hypocotyls displayed a gradually shrinkage whereas WT hypocotyls resisted better to the induced desiccation (*n* = 11). Hypocotyl diameters were measured using the ImageJ software. The results were analyzed by ANOVA and Tukey HSD test (α = 0.01) using R software. Different letters indicate significant differences at *p*-value < 0.01. **(C)** Permeability of etiolated hypocotyls was examined using the TB staining test. TB uptake was quantified spectrophotometrically at OD_626__nm_. Results are presented as mean values ± SD (*n* = 5). Significant differences from WT were assessed by a Student’s *t*-test (^∗∗^*p*-value < 0.01).

According to (i) the putative function of nsLTPs, and (ii) the above-demonstrated expression profile of *AtLTP2* restricted to the aerial organ epidermal cells, we have performed cuticle-dedicated phenotyping experiments. Given that a crucial function of the cuticle is its role as a primary barrier to water-loss, the cuticle permeability was determined in 5-day-old etiolated seedlings of WT, *atltp2-1*, and *atltp2-1* (comp) seedlings. At this developmental stage, following *AtLTP2* expression peak (**Figure 1B**), AtLTP2 has been shown to be strongly accumulated in etiolated hypocotyl cell walls by previous proteomic studies (Irshad et al., 2008). The water-loss rate of the etiolated seedlings was faster in *atltp2-1* than in WT following exit from the *in vitro* conditions operated in order to mimic a desiccation stress by quickly reducing the *in vitro* water-saturated atmosphere (**Figure 5A**). The water-loss rates of the 5′2 and 14′3 complemented lines were slightly slower than the WT one (**Figure 5A**). A link was found between relative *AtLTP2* transcript level and the water-loss rate: the higher the *AtLTP2* transcript level is (Supplementary Figure S1), the more the seedlings resist to water-loss (**Figure 5A**). To ensure that the observed weight differences were due to water-loss, the dry mass of the seedlings of the different genotypes was measured. No significant difference was detected between them (**Figure 5A**). The observed faster desiccation in *atltp2-1* etiolated seedlings could be visualized after exit from *in vitro* conditions (**Figure 5B**). Although both the WT and *atltp2-1* hypocotyls displayed similar diameters at the time they were put in normal atmospheric conditions, a clear shrinkage quickly appeared in *atltp2-1* hypocotyls, whereas the WT ones resisted better to dehydration during the time course of experiments (**Figure 5B**). These observations were confirmed by biometrical data showing that *atltp2-1* hypocotyl diameters became significantly smaller than the WT ones a few minutes after their transfer to normal atmospheric conditions (**Figure 5B**).

Moreover, non-functional cuticle often showed an increased permeability to water-soluble molecules that can be monitored with toluidine blue (TB) hydrophilic dye uptake (Tanaka et al., 2004). The *atltp2-1* hypocotyls showed an intense TB coloration (**Figure 5C**) as compared to those of the WT and of the two selected complemented lines which remained mostly white. Toluidine blue was extracted for quantification from stained aerial organs. Significant 6- to 10-fold higher stain intensity was revealed for *atltp2-1* seedlings as compared to WT and complemented lines, in agreement with the macroscopic observations (**Figure 5C**).

**FIGURE 6:**
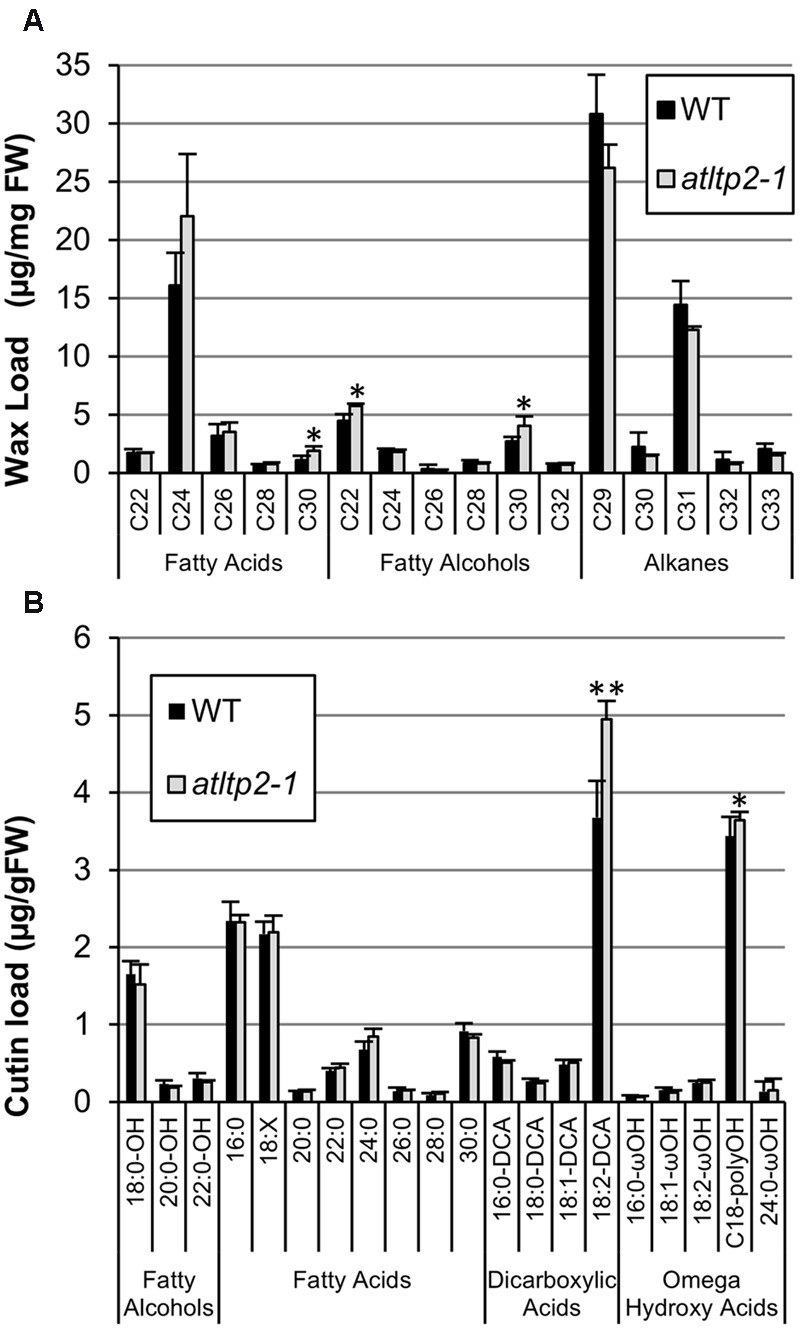
***atltp2-1* and WT etiolated hypocotyls have very similar cuticle composition and content.** Cuticular waxes **(A)** and cutin monomers **(B)** were quantified by GC-MS. The different aliphatic compounds, sorted into individual chain length, are presented. Mean values are shown in μg/mg of fresh weight (FW) ± SD (*n* = 4). 18:X stands for the sum of all unsaturated C18 fatty acids (18:1+18:2+18:3). Significant differences were assessed by a Student’s *t*-test (^∗^*p*-value < 0.05 and ^∗∗^*p*-value < 0.01).

In order to determine if this permeability phenotype was linked to defects in the cuticle, the composition and content of the cuticular waxes and cutin polymer were determined by GC-MS using 5-day-old etiolated hypocotyls. To our knowledge, no work has yet reported the cuticle composition of dark-grown seedlings. Therefore, the cuticle lipid profiles obtained were compared to those of light-grown *A. thaliana* stems and leaves (Suh et al., 2005; Kosma et al., 2009). The wax profile of etiolated hypocotyls was dominated by C29 and C31 alkanes, like in leaves, but also contained substantial amounts of very long-chain fatty acids with less than 28 carbon atoms (mainly C24) and very long-chain fatty alcohols with less than 26 carbon atoms (mainly C22; **Figure 6A**). Such compounds were found as very minor components of *A. thaliana* leaf and stem waxes, which usually contained similar compounds (i.e., fatty acids and alcohols), but with more than 26 carbon atoms. Moreover, *A. thaliana* stems were characterized by high amounts of C29 ketone and secondary alcohols, which were not detected in hypocotyls (**Figure 6A**). The cutin polymer of etiolated hypocotyls was dominated by 18:2 dicarboxylic acid, as in leaves and stems, but also contained high amounts of polyhydroxy C18 fatty acids (**Figure 6B**). In addition, very long-chain fatty acids with more than 24 carbon atoms were detected. Nevertheless, the waxes and cutin polymer composition and content of WT and *atltp2-1* presented only slight differences (**Figure 6**). The waxes profile of *atltp2-1* was characterized by very minor increases in the amounts of C30 fatty acids, and C22 and C30 fatty alcohols, while the major alkanes (C29 and C31) were not significantly decreased (**Figure 6A**). More interestingly, at the cutin level, *atltp2-1* was characterized by a one third increase of the major component (18:2-DCA) when compared to WT (**Figure 6B**). These analyses therefore suggested that alterations in the cutin polymer, rather than in the cuticular waxes, could be related to the observed increased permeability.

### *atltp2-1* Displays a Collapsed Epidermal Cell Surface and a Disrupted Ultra-Structure at the Cuticle-Cell Wall Interface

**FIGURE 7:**
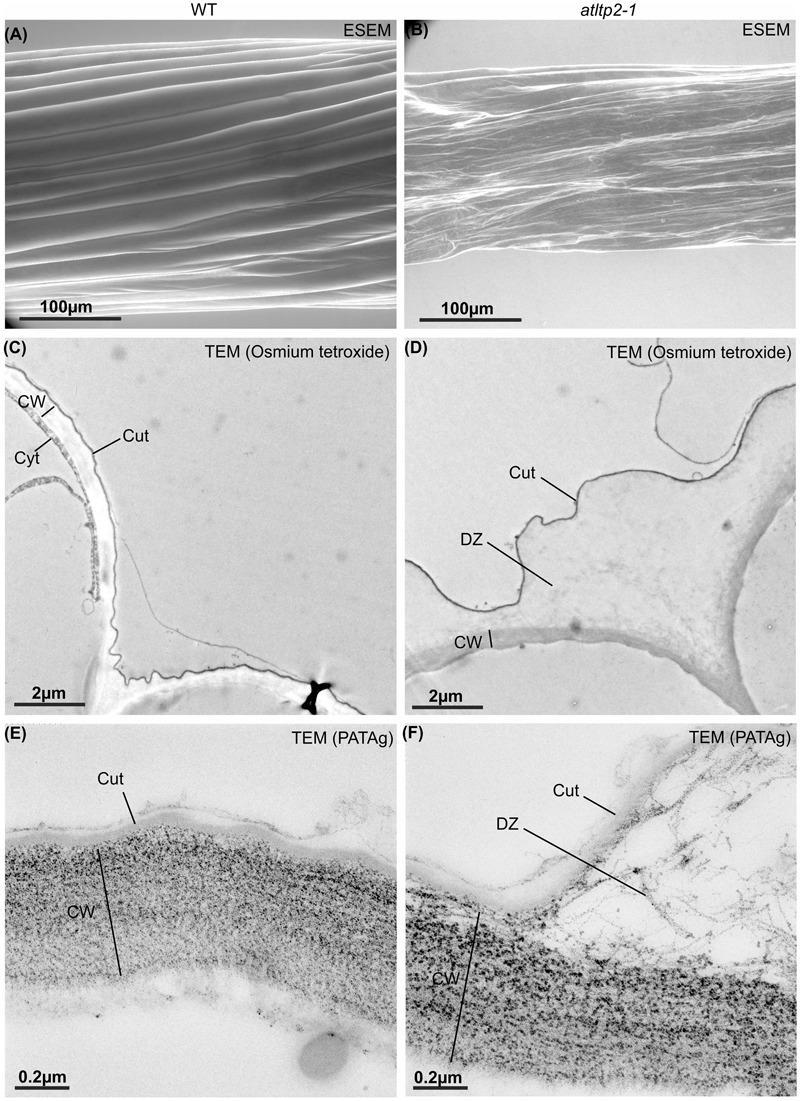
**Etiolated hypocotyls of *atltp2-1* show dramatic surface and cuticle-cell wall interface defects.** Hypocotyls from 5-day-old WT **(A,C,E)** and *atltp2-1* **(B,D,F)** etiolated seedlings were imaged using ESEM **(A,B)**, osmium tetroxide lipophilic staining at the transmission electron microscopy (TEM) level **(C,D)**, and PATAg cell wall polysaccharide staining at the TEM level **(E,F)**. Note the dramatic perturbation of the *atltp2-1* hypocotyl surface imaged by environmental scanning electron microscopy (ESEM). Legend: cuticle (Cut); cytoplasm (Cyt); cell wall (CW); cuticle-cell wall detachment zone (DZ).

To clarify which alteration in the cuticle and/or cell wall could result in the permeability phenotype of *atltp2-1*, the epidermal cell surface and ultra-structure of etiolated hypocotyls were investigated by electron microscopy techniques. Environmental scanning electron microscopy (ESEM) was used to have an overview of the hypocotyl surface under close-to-native conditions. ESEM was indeed particularly adapted to the observation of the delicate hypocotyls because no fixation of the samples was required, thus preventing/limiting dehydration effect. While WT hypocotyls showed extremely elongated epidermal cells evenly aligned, the architecture of the *atltp2-1* epidermal cells displayed pronounced modifications both in the shape and cell organization leading to some points of disruption along their surface (**Figures 7A,B**). This collapsed phenotype was observed from 3 to 11 days post-germination for *atltp2-1* (Supplementary Figure S5), whereas a WT phenotype was repeatedly observed for the three complemented lines (Supplementary Figure S6). Whether these different phenotypes actually corresponded to the native state of the hypocotyls or were accentuated by the vacuum during ESEM, they clearly illustrated an impaired hypocotyl cell surface in *atltp2-1*.

**FIGURE 8:**
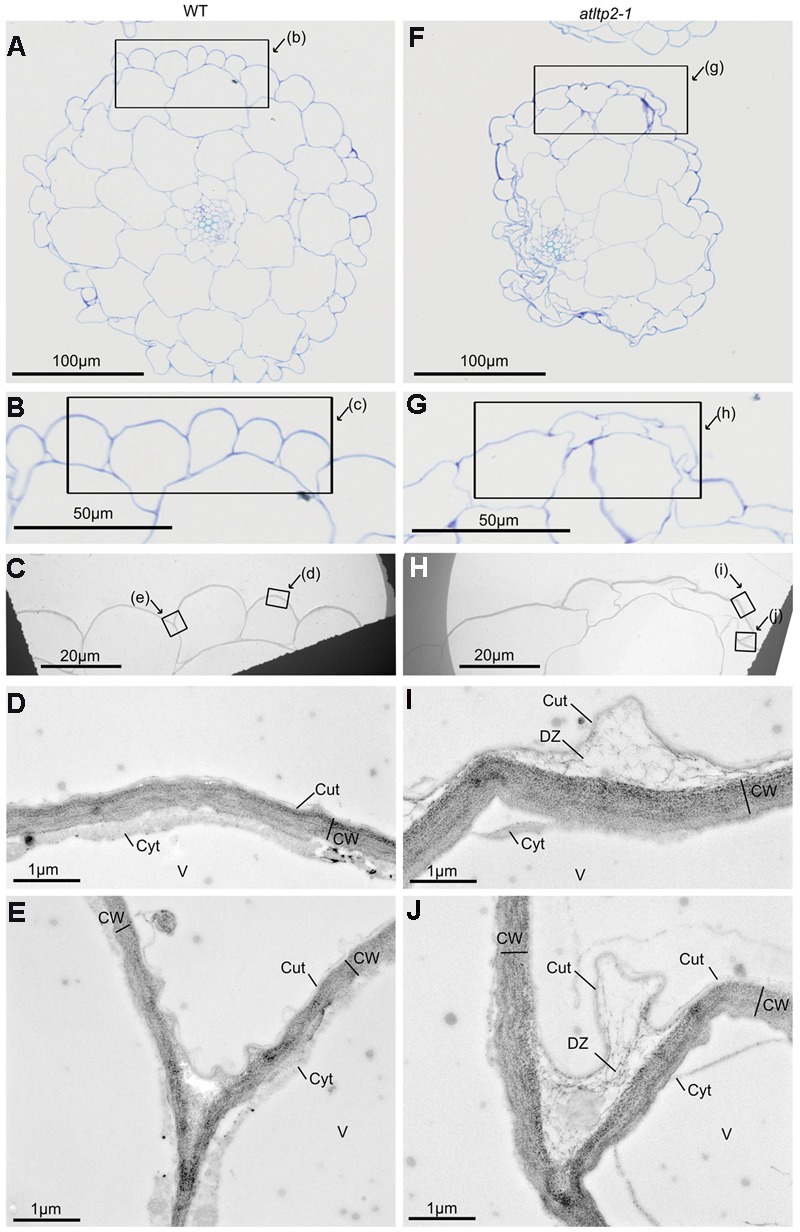
**Correlated light microscopy (LM) and TEM observations of 5-day-old etiolated hypocotyls of WT and *atltp2-1*.** Semi-thin cross-sections of WT **(A–E)** and of *atltp2-1* **(F–J)** were stained with TB for LM observations **(A,B,F,G)** and ultra-thin serial sections were PATAg-labeled for TEM observations **(C–E,H–J)**. The frames indicate the magnified zones. Note the overall shrinkage of *atltp2-1* hypocotyl, although no particular phenotype was observable at the cuticle-cell wall interface at the correlated high magnification of LM **(B,G)** and low magnification of TEM **(C,H)**, respectively. In the *atltp2-1*, higher TEM magnification **(I,J)** allowed, the visualization of the PATAg-reactive cell wall defibrillated material at the detachment zone located between the cuticle and the cell wall. Legend: cuticle (Cut); cytoplasm (Cyt); cell wall (CW); cuticle-cell wall detachment zone (DZ); vacuole (V).

The ultra-structure of both epidermis periclinal cell wall and cuticle was observed by transmission electron microscopy (TEM) using the osmium tetroxide lipophilic staining (**Figures 7C,D**) and the PATAg polysaccharide staining (**Figures 7E,F**). A typical ∼50 nm thick electron-dense layer corresponding to the cuticle was observed on the outer part covering the periclinal cell wall in hypocotyl cross-sections from both WT and *atltp2-1* (**Figures 7C,D**). However, the *atltp2-1* cuticle was clearly no longer sealed to the cell wall at several points, especially at the junction zone between two cells, thus delineated detachment zones (DZ) between the *atltp2-1* cuticle and the underlying periclinal cell wall. On the contrary, the WT cuticle consistently appeared attached to the cell wall (**Figures 7C,D**). While the PATAg strongly stained both the WT and the *atltp2-1* epidermis cell walls (**Figures 7E,F**), the DZ were lightly and specifically stained by PATAg, but not by osmium tetroxide (**Figures 7C,D**), indicating that they were mainly composed of fibrillar cell wall polysaccharides which were pulled up during the cuticle-cell wall separation. This repeatedly observed phenotype was reinforced by a quantitative analysis of the DZ area stained with PATAg, at the junction zone between two cells. The ratio of detached cuticle length normalized relatively to the length of outer periclinal cell wall was significantly higher in *atltp2-1* (75% +/- 8.8 %) than in WT (14.6% +/- 5.9 %; Supplementary Figure S7). Furthermore, light microscopy (LM) observation of cross-sections confirmed the remarkable impaired morphology of *atltp2-1* hypocotyls in which the epidermal cells were particularly collapsed and thereby modified the overall shape of hypocotyls (**Figure 8**). By performing correlated LM and TEM-PATAg polysaccharide staining, it was shown that the DZ were distributed along the hypocotyl surface and were indeed made of empty space with little cell wall material, especially at the position covering the epidermal radial cell wall (**Figure 8**). Altogether, these results have demonstrated marked ultra-structural alterations on the epidermal surface of the *atltp2-1* etiolated hypocotyls, suggesting a role for AtLTP2 at the cuticle-cell wall interface.

## Discussion

### In Etiolated Seedlings, the Abundance of *AtLTP2* Gene Products Is Restricted to Cuticle-Carrying Epidermal Cells

The rationale to study AtLTP2 was primarily linked to the fact that it was the unique and abundant member of the nsLTP multigene family present in the cell wall proteome of etiolated hypocotyls (Irshad et al., 2008). In agreement with the *AtLTP2* expression patterns described in the *Arabidopsis* eFP browser database (Winter et al., 2007), we found that the expression of *AtLTP2* was 15–30 times increased over a 7 days period in the dark. In addition, we have demonstrated here that in etiolated seedlings, the *AtLTP2* strong expression was restricted to epidermal cells of hypocotyls and to a lesser extent of cotyledons. In contrast, when *atltp2-1* plants were grown under a 16 h light/8 h dark photoperiod in soil in a greenhouse to produce seeds, we did not notice any obvious change in germination rate, early vegetative growth, flowering time, and seed production in comparison to WT (data not shown), suggesting low expression under normal conditions. In agreement, using transgenic *AtLTP2* promoter::GUS lines, Chae et al. (2010) previously showed that *AtLTP2* was only expressed at a low level in hypocotyls in normal growth conditions, but highly induced under abiotic stress such as drought (PEG treatment). More recently, using the same strategy, Deeken et al. (2016) reported that *AtLTP2/AtLtpI-4* expression was restricted to very specific cell types under normal conditions, but reached very high level in crown gall tumors which develop on inflorescence stems epidermis of mature plants upon bacterial infection. Finally, abundant *AtLTP2* transcripts were also found in the epidermal cells of embryonic aerial organs at late stages of seed development (Francoz et al., 2016). Taken together, these different reports showed that *AtLTP2* was poorly expressed in most plant organs under normal exposure, but strongly expressed in WT aerial organ epidermis undergoing cell wall remodeling. Interestingly, the *AtLTP2* developmental expression pattern is in addition distinguishable from those of other *AtLTP*s already studied (Winter et al., 2007; Chae et al., 2010), allowing to hypothesize that AtLTP2 could play a major role in different scenarii related to biotic (crown gall) and abiotic stresses (drought/PEG, continuous darkness/active growth).

### AtLTP2 Is Transported through the Secretory Pathway Both to the Cell Wall and the Plastids

As expected from AtLTP2 identification in the cell wall proteome of etiolated hypocotyls (Irshad et al., 2008), we unambiguously visualized over-expressed AtLTP2-TagRFP in the cell wall compartment of *N. benthamiana* epidermal cells. Unexpectedly, AtLTP2 fused with the red fluorescent protein mCherry was reported as being associated with the plasma membrane (Deeken et al., 2016) despite the absence of GPI anchor necessary for plasma membrane-anchorage (Debono et al., 2009; Lee et al., 2009; Kim et al., 2012). We hypothesize that this discrepancy could be due to the plasmolysis treatments done with 1 M KNO3 (Deeken et al., 2016) or mannitol (this study). Indeed, we have previously shown that salt solutions and not osmotic agents such as glycerol or mannitol, release fluorescent-tagged CWPs from cells walls (Albenne et al., 2014). Furthermore, AtLTP1/AtLtpI.5 (*At2g38540*), a nsLTP highly homologous to AtLTP2 (81% protein similarity), was demonstrated to have a cell wall localization (Thoma et al., 1993; Potocka et al., 2012; Deeken et al., 2016). Altogether and considering the huge abundance of AtLTP2 in the cell wall proteome of etiolated hypocotyls, these results provide strong clues in favor of its cell wall localization. Other nsLTPs from several plant species have already been shown to be addressed to the apoplastic compartment [see for review (Liu et al., 2015)]. Interestingly, nsLTPs were also found deeply associated with the surface wax (Pyee et al., 1994), the cutin layer (Thoma et al., 1993). Our localization experiments did not allow showing whether AtLTP2 was localized to the cuticle, to the underlying polysaccharidic cell wall or both. However, the sample preparation procedure used for the cell wall proteomics studies in which AtLTP2 was abundantly found, argues for cell wall localization (Irshad et al., 2008). Surprisingly, we consistently additionally detected AtLTP2-TagRFP and AtLTP2-YFP in plastids of *N. benthamiana* leaves during transient expression assays. Although AtLTP2-mCherry was not detected in this sub-cellular compartment (Deeken et al., 2016), the plastidial localization of both AtLTP2-TagRFP and AtLTP2-YFP was in agreement with the identification of AtLTP2 but also AtLTP1 in the *A. thaliana* chloroplast proteome (Ferro et al., 2010). It is worth noting that during somatic embryogenesis in *A. thaliana*, LTP1 epitopes (i.e., AtLTP1 and/or AtLTPs sharing protein similarity with AtLTP1 as AtLTP2), were strongly and clearly detected in plastids (Potocka et al., 2012). Furthermore, the *Rlem*LTP from rough lemon was also localized restrictively to the chloroplast but its principal role remains unknown (Nishimura et al., 2008). Altogether, AtLTP2 appears to be localized both to the cell wall and the plastids. In this study, our results suggest that this dual targeting involves the secretory pathway. Indeed, the transport of AtLTP2 to the cell wall and to plastids was prevented in presence of BFA, a fungal molecule mainly reported as an inhibitor of anterograde trafficking in plants even if in some cases it could also inhibits endocytosis (Jelínková et al., 2015). Recently, three rice Nucleotide Pyrophosphatase/Phosphodiesterases (NPP1, 2 and 6) have been shown to be transported from the secretory pathway to plastids *via* the *trans*-Golgi. In particular, their transport to plastids was BFA-sensitive (Nanjo et al., 2006; Kaneko et al., 2016). Only a few proteins have been shown to be targeted to the plastids through the secretory pathway, e.g., NPPs, several α-amylases and a carbonic anhydrase (Radhamony and Theg, 2006; Li and Chiu, 2010; Kaneko et al., 2016). AtLTP2 could then be added to the shortlist of proteins targeted, through the secretory pathway, to non-secretory pathway organelles such as plastids, mitochondrion, or nucleus (Porter et al., 2015).

### AtLTP2 Is Required to Maintain the Cuticle-Cell Wall Interface Integrity to Control Etiolated Hypocotyl Permeability

Plant epidermal cells have a huge demand for lipid synthesis and lipid transport as they synthesize the cuticle that covers their surface (Hurlock et al., 2014). Because of their lipophilic properties, the cuticular components may require the use of transporters for their trafficking from the ER to the cuticle. nsLTPs were proposed as such carriers for cuticular lipids through the hydrophilic cell wall compartment (Kader, 1996; Samuels et al., 2008; Domínguez et al., 2015). According to its epidermal localization and to its putative role in cuticle formation, AtLTP2 was assessed for its involvement in cuticle permeability. *atltp2-1* knock-down mutant displayed a faster water-loss rate together with an increase in TB staining revealing deep modification in the permeability and possibly in the structure of the cuticle. In *in vitro*-grown etiolated hypocotyls, no stomata were observed on our ESEM images from all genetic lines strongly suggesting that the water-loss measured mainly occurred at the cuticular level. The dysfunctional cuticle observed in etiolated *atltp2-1* seedlings was entirely recovered in complementation lines. However, *atltp2-1* and WT etiolated hypocotyls displayed very similar cuticle composition and content. It is considered that distortion of the cuticle can result in an increased permeability (Tanaka et al., 2004) which in many instances, is linked to modifications in the cuticle composition and content. For instance, mutations in ABC transporter encoding genes (Luo et al., 2007; Bessire et al., 2011), in *BODYGUARD* (*BDG*) encoding an α/β-hydrolase fold protein (Jakobson et al., 2016), or in lipid biosynthetic enzymes involved in cuticle formation (Tanaka et al., 2004; Kosma et al., 2009; Li et al., 2013), were tightly associated with changes both in cuticle permeability and cuticle composition and content. However, very few reports have clearly demonstrated direct links between nsLTPs and cuticular permeability and/or cuticle lipid profile. Overexpression of *BraLTP1* in *Brassica napus* (Liu et al., 2014) causes both reduced wax deposition and a significant increase in water-loss. In addition, the disruption of genes encoding plasma membrane-anchored LTPG reported to be implicated in cuticular lipid composition did not lead to changes in cuticle permeability and show a slight impact on lipid content and composition (Lee et al., 2009). Our cuticular lipid analyses showed that *atltp2-1* was slightly affected in its cutin polymer. Besides, the cuticular waxes were barely affected, although they play the predominant role in limiting non-stomatal water-loss (Jetter and Riederer, 2016). Interestingly, in accordance with these results, in the physiological context of the crown gall, the GK-639E08 *atltp2* mutant displayed no difference in lipid composition of the cuticular wax in comparison with WT (Deeken et al., 2016). Thus, AtLTP2 appears to be a new type of nsLTP which could be involved in the barrier primary function of the cuticle by limiting water-loss at the epidermal surface of etiolated hypocotyls without major modification in its wax cuticular lipid profile.

To go further in the understanding of the role of AtLTP2 in controlling cuticular permeability, the structure and ultra-structure of the surface of etiolated hypocotyls were examined. When *AtLTP2* expression was strongly reduced, the epidermal cells appeared disorganized and several points of disruption along the hypocotyl surface were observed. Severe ultra-structural alterations at the cuticle-cell wall interface occurred in *atltp2-1* leading (i) to the detachment of the cuticle from the underlying cell wall mainly at the radial separation of epidermal cells and (ii) to the appearance of space with remaining cell wall components. It cannot be excluded that both the vacuum and dehydration treatments applied for ESEM and TEM, respectively, could have accentuated the mutant phenotype. However, this phenotype was exclusively observed in *atltp2-1* and not in WT or in complemented lines. It indicated that the cuticle-cell wall interface was highly weakened in *atltp2-1* in comparison to WT and complemented lines. Besides, no phenotype was observed in the ultra-structure of the cuticle itself.

The two observed phenotypes in *atltp2-1* (increase of permeability and alteration of cuticle-cell wall interface integrity) without strong modification of the cuticle composition, content, and ultra-structure, do not fit with the historical purported role of nsLTPs (Kader, 1996; Samuels et al., 2008). Indeed, nsLTPs have long been assumed to shuttle lipids across the hydrophilic cell wall from the plasma membrane to the cuticle. Given the abundance and the uniqueness of AtLTP2 in the studied model, and the phenotype observed in *atltp2-1*, one would expect *atltp2-*1 to display cuticle composition/content and ultra-structure phenotypes, if AtLTP2 would have such a shuttling role. Such relationships between ultra-structural and/or biochemical changes within the cuticle and increased permeability were described in different plant species mutants thought to be defective in the biosynthesis of cuticular compounds (Voisin et al., 2009; Mao et al., 2012; Liu et al., 2014). On the contrary, our results indicate that AtLTP2 could be involved, directly or indirectly, in the integrity of the cuticle-cell wall adhesion in etiolated seedlings to maintain the cuticle barrier properties, but without disturbing the cuticle ultra-structure itself. Two hypotheses could be proposed to explain the AtLTP2 action mode: (i) AtLTP2 could be a carrier of cuticular lipids and its down-expression leads to slight modifications of cuticle composition and ultra-structure (ii) AtLTP2 is not involved in cuticle formation, but rather could play a physical role at the cuticle-cell wall interface. In our opinion, the fact that the observed slight modifications in cuticle composition did not affect the cuticle ultra-structure in *atltp2-1*, and the abundance of AtLTP2 are not in favor of a role as a carrier for cuticular compounds. It rather suggests that AtLTP2 could play a structural role as an amphiphile molecule with adhesive properties that could allow the accurate sealing between the hydrophobic cuticle and the hydrophilic underlying cell wall. This proposed structural role is strengthened by the spatio-temporal localization of *AtLTP2* gene products (Irshad et al., 2008; our results). Even though we were not able to determine the localization of AtLTP2 within the cell wall, the observed ultra-structural phenotype suggests that AtLTP2 may localize at the cuticle-cell wall interface where intra-cuticular waxes are packed in the spaces of the cutin polyester, acting together to maintain the permeability barrier (Goodwin and Jenks, 2005; Bessire et al., 2007). Somehow similar phenotype with the loss of sealing at the cuticle-cell wall interface were observed for mutants of plasma membrane-localized ABC transporters implicated in cutin deposition like *AtABCG32* (Bessire et al., 2011) and *OsABCG31* (Garroum et al., 2016). Given that the cuticle layer which directly interacts with the cell wall is rich in cutin residues (Yeats and Rose, 2013), we assume that cutin could be bound by AtLTP2, constituting a first level of AtLTP2 structural interaction at the cuticle-cell wall interface.

A second level of hypothesized AtLTP2 structural interactions concerns polysaccharides. The intimate linkage between both polysaccharides and lipid cuticle constituents are proposed to have a drastic impact on cuticle water absorption (Bessire et al., 2007; Fernandez et al., 2016). However, the molecular basis of lipid-polysaccharide interactions is unclear. Based on phylogenetic analysis combined with ESI (Electrostatic Similarity Indice) clustering analysis (Chae et al., 2010), AtLTP2 belongs to the Stigma/style Cysteine-rich Adhesin (SCA-like LTP) positively net charged subset and was closely clusters with the tobacco LTP2 (Chae et al., 2010). Thus, we propose that, in addition to AtLTP2 interaction with hydrophobic lipids in its pocket, the positive electrostatic polarization of AtLTP2 surface could allow additional interaction with negatively charged cell wall polysaccharides such as demethylated homogalacturonans (Chae et al., 2010). Indeed, it was assumed that during pollen tube adhesion-mediated guidance, the positive lily SCA-LTP interact with the negative pectin moieties from the stylar extra cellular matrix (Mollet et al., 2000), then leading to the adhesion of the pollen tube by the formation of an adhesive matrix, due to ionic interactions between the three partners, SCA-LTP, the pectins of both pollen tube walls and stylar surfaces of the transmitting tract epidermis (Mollet et al., 2007). No such interaction has yet been demonstrated for other nsLTPs, but interaction with cellulose has also been proposed for a tobacco nsLTP presenting a cell wall-loosening activity dependent on hydrophobic interactions (Nieuwland et al., 2005).

To conclude, we propose that AtLTP2 contributes to the cuticular main function (e.g., a barrier to non-stomatal water-loss) by preserving the integrity of the sealing/adhesion at the cuticle-cell wall interface in epidermal cells of dark-grown aerial organs. Given its abundance, its amphiphilic nature and the observed phenotypes of *atltp2-1*, we propose a structural role for AtLTP2 at this interface made of lipidic cuticular compounds and hydrophilic polysaccharides, constituting a novel function for a nsLTP. Interestingly, during growth and expansion of crown gall tumors in which *AtLTP2* was highly expressed (Deeken et al., 2016), the epidermal cell layer was disrupted, leading to the loss of an intact cuticule (Gohlke and Deeken, 2014). Thereby, the disrupted crown gall surface has to be sealed and suberization of cell walls most probably occurred to avoid uncontrolled loss of water from the host plant. Similarly, Chae et al. (2010) reported strong *AtLTP2* expression in response to PEG treatment, suggesting the involvement of AtLTP2 in osmotic response, where the cuticular barrier is known to be up-regulated (Kosma et al., 2009). Thus, AtLTP2 could present different functions in various developmental conditions or under biotic or abiotic stresses. Remarkably, in all three cases (high elongation rate of etiolated hypocotyls, biotic and abiotic stresses), it seems that the expression of *AtLTP2* is induced in specific conditions where reinforcement of the cuticle-cell wall integrity or modification of the cell wall (e.g., suberization) is needed at the epidermal surface.

In addition, AtLTP2 is the first nsLTP shown to have a dual sub-cellular localization to the cell wall and the plastids by trafficking through the secretory pathway. The elucidation of the molecular mechanisms by which AtLTP2 establish cell wall-cuticle homeostasis and the exact function of the dual targeting will be challenging tasks in the future.

## Materials and Methods

### Plant Material

The *atltp2-1* mutant for *At2g38530* (Salk N526257.5, Col-0 background) was ordered from the NASC collection^1^. The homozygous *atltp2-1* and its WT counterpart were obtained from this Salk line during a genotyping process of its progeny. This screening was done by PCR with the primers listed in Supplementary Table S1. For phenotyping experiments, seeds were sown in Magenta boxes on Murashige and Skoog (MS) medium (Sigma–Aldrich)^2^ with 1% (w/v) agar. Seeds were kept in the dark at 4°C during 48 h and subsequently exposed to light for 4 h to synchronize germination. *In vitro* seedlings were grown in the dark at 23°C for various periods of time up to 11 days. For complementation/transformation experiments, *atltp2-1* plants were grown in growth chamber under a 15 h light/9 h dark cycle (22/20°C, 70% humidity) until flowering. For transient transformation experiments, *N. benthamiana* plants were cultivated in growth chambers (16 h light/8 h dark cycle, 25/22°C, 80% humidity) during 3 weeks.

### Plasmid Construction and Stable Plant Transformation of *atltp2-1*

A 2.8 kb genomic fragment comprising the *AtLTP2* promoter, the coding region and the terminator regions was amplified from genomic DNA by PCR using primers listed in Supplementary Table S1. It was cloned into pAM-PAT-D35S-GWY-3HA-RRS1-RT which derived from binary vectors previously described (Tasset et al., 2010), to generate the prom*AtLTP2::AtLTP2::*Term*AtLTP2* construct. The *Agrobacterium tumefaciens* GV3101::pMP90 strain (Koncz and Schell, 1986) was transformed with the recombinant vector and used to stably transform *atltp2-1* plants with the floral dip method (Clough and Bent, 1998).

### *In situ* Hybridization (ISH)

*In situ* hybridization was performed as previously described for developing seeds (Francoz et al., 2016). Tissue microarrays encompassed early developmental kinetics for hundreds of etiolated seedlings of *A. thaliana* WT, *atltp2-1*, and the complemented line 5′2. Slides have been scanned with a Nanozoomer HT slide scanner (Hamamatsu)^3^.

### RNA Extraction and cDNA Synthesis

Total RNA extractions were performed from three biological replicates for each sample. For the youngest samples, whole seedlings were sampled, whereas for 5- and 11-day-old seedlings, only aerial organs were sampled. Samples were ground in liquid nitrogen and RNA extraction was performed using the E.Z.N.A^®^ Plant DNA Kit (Omega BioTech Inc)^4^. For each extraction, 70 mg of fresh material were used. RNA concentration and quality were determined using a N-100 NanoDrop^5^. For cDNA synthesis, 400 ng of total RNAs were used to perform reverse transcription using the High-Capacity cDNA reverse transcription kit (Applied Biosystems)^6^. The cDNAs were stored at -20°C.

### RT-qPCR Analysis

RT-qPCRs were laid out in 384 well-plates, including three reference genes (*At5g15710* encoding a F-box protein, *At1g49240* encoding actin 8 and *At4g34270* encoding a TIP-41 like protein; Czechowski et al., 2005). *A. thaliana* gene-specific primers are listed in Supplementary Table S1. Three biological and two technical replicates were included for each cDNA-primer combination. Individual reactions contained 3 μl of cDNA, 0.5 μl of each primer (10 μM), and 4 μl of 2x SYBR GREEN^TM^ (Roche)^7^ in a final volume of 8 μl. Plates were sealed using a clear, adhesive PCR film (Thermo Scientific)^8^. They were run in a LightCycler^®^ 480 (Roche) at 95°C for 5 min, followed by 45 cycles with 95°C for 15 s and 60°C for 1 min, and finally 40°C for 1 min. Data were collected and analyzed using Microsoft Excel 2010^9^. The geometric mean value generated from the three reference genes was used to calculate the relative gene expression levels using the 2^-ΔΔCT^ method (Livak and Schmittgen, 2001). Significant differences were assessed by a Student’s *t*-test.

### Cell Wall Purification, Extraction of Proteins, and Identification of AtLTP2 by MALDI-TOF MS

Cell wall purification and extraction of proteins were performed as described (Feiz et al., 2006) with 5 g of etiolated hypocotyls and cotyledons. Proteins were dialysed and concentrated by successive centrifugation using the Amicon Ultra-15 centrifugal filter Ultracel^®^ (Merck Millipore Corporation)^10^. Proteins were separated by denaturing 1D-E using 12.5% (m/v) polyacrylamide gel. Proteins were stained with Coomassie^TM^ Brilliant Blue (Scheler et al., 1998). Colored bands around 9.6 kDa were sampled and digested with trypsin. MALDI-TOF MS analyses were performed as reported (Boudart et al., 2005).

### Transient Transformation of *N. benthamiana* Plants for Sub-cellular Localization of AtLTP2

To generate the *AtLTP2-TagRFP* construct, the *A. thaliana* full-length cDNA *AtLTP2* clone (pda02841) was ordered at the RIKEN Genomic Sciences Complex (RIKEN^11^; Seki et al., 2002). It was amplified by PCR using the primers listed in Supplementary Table S1 (AttB1-At2g38530-F and Spacer-At2g38530-R). The *TagRFP* coding sequence was amplified separately from the pTagRFP-C vector (FP141, Evrogen, Euromedex, Souffelweyersheim, France) with the sense and AS primers (Spacer-TarRFP-F and AttB2-TagRFP-stop listed in Supplementary Table S1). Both the *AtLTP2 and the TagRFP* PCR products were fused by the overlap extension PCR method and the corresponding fused DNA product was amplified with the primers listed in Supplementary Table S1 (attB1-F-adapter and attB2-R-adapter). Then, it was cloned into the entry vector pDONR207 prior to be transferred into the pEAQ-DEST1-HT destination vector (Sainsbury et al., 2009) by homologous recombination using the Gateway^®^ technology (Invitrogen^TM^-ThermoFisher)^12^. The *A. tumefaciens* GV3101 strain was transformed with the recombinant binary vector.

*Agrobacterium tumefaciens* were grown in Yeast Extract Broth (YEB) liquid medium at 28°C under shaking until OD_600_ reached 1. Bacteria were centrifuged 5 min at 4000 × *g*. The pellet was re-suspended in agromix (10 mM MES-KOH pH 5.6, 0.15 mM acetosyringone, 10 mM MgCl_2_) until OD_600_ = 1, and incubated overnight at room temperature. *Nicotiana benthamiana* leaves of 3-week-old plants were infiltrated at their abaxial side with the bacterial suspension(s). In the case of co-infiltration of two bacterial suspensions carrying different constructs, a ratio of 2/1 was used [TagRFP-construct/YFP-tagged organelle markers for plasma membrane (pm-yb CD3-1006) or ER (ER-yb CD3-958)] (Nelson et al., 2007). *Nicotiana benthamiana* plants were kept in the growth chamber room until observation 48 h after infiltration.

An upright confocal laser scanning microscope (LEICA SP2 AOBS^13^) using a 40 x apochromatic water immersion lens was used to visualized YFP fluorescence (excitation: 488 nm; emission: 514–551 nm), TagRFP fluorescence (excitation: 561 nm; emission: 582–622 nm), and chlorophyll auto-fluorescence (excitation: 488 nm; emission: 680–720 nm). Fluorescence in different channels was acquired for the same field using a sequential acquisition mode. Plasmolysis assays were performed using 0.5 M mannitol.

For the ER-Golgi trafficking assay, BFA (Sigma–Aldrich) was used at a concentration of 35 μM. One milliliter BFA was co-infiltrated with transformed *A. tumefaciens* in *N. benthamiana* leaves and 1 ml was infiltrated 24 h before observation (Daskalova et al., 2010).

### Water-Loss and Toluidine Blue Assays

Five-day-old etiolated seedlings from WT and *atltp2-1* (*n* = 11) were aligned on a microscopy slide and imaged with an Axiozoom V 16^14^ during a 25 min kinetics to follow their degree of desiccation. Hypocotyl diameters were measured using the ImageJ software^15^. The results were analyzed by ANOVA and Tukey HSD test (α = 0.01) using the R software (R Core Team, 2016). In parallel, 500 mg of WT, *atltp2-1*, and complemented lines (5′2 and 14′3) seedlings were put in Petri dishes. They were placed in a 25°C oven and weighted with a precision balance during a 3 h kinetics to monitor water-loss. Dry mass was measured after 3 days at 25°C. Significant differences were assessed by a Wilcoxon rank-sum test using the R software. TB staining was performed as described (Tanaka et al., 2004). Quantification of TB was performed using 20 mg of etiolated hypocotyls (Xing et al., 2013). Significant differences were assessed by a Student’s *t*-test. Root and seed coats were carefully removed prior to immersion in ethanol.

### Wax and Cutin Monomer Analysis

Five-day-old etiolated hypocotyls were harvested, the cotyledons removed, and the fresh mass immediately measured. Waxes were extracted by dipping in chloroform for 30 s and analyzed as described (Bourdenx et al., 2011). The hypocotyls were then delipidated and the cutin monomer composition and content determined as described (Domergue et al., 2010). Significant differences were assessed by a Student’s *t*-test.

### Environmental Scanning Electron Microscopy Analysis

Wild type and *atltp2-1* etiolated seedlings were used for ESEM analysis. Samples were observed without fixation following their sampling from *in vitro* cultures. Micrographs were obtained with a Quanta 250FEG ESEM (FEI Company) ^16^ using a 10 kV acceleration voltage, a spot size of 3, and a chamber pressure of 800 Pa, at 5°C and 94% humidity.

### Transmission Electron Microscopy Analysis

Five-day-old etiolated seedlings of WT and *atltp2-1* were fixed for 2 h in 1.25% glutaraldehyde/2% paraformaldehyde (Oudin et al., 2007). Half of the samples were post-fixed for 1 h in 1% (v/v) aqueous OsO_4_ solution (Electron Microscopy Sciences)^17^. All the samples were further dehydrated, infiltrated, and embedded in LR White resin (Oudin et al., 2007). For OsO_4_ post-fixed samples, ultrathin cross-sections (100 nm thick) of the hypocotyls base were cut and directly observed on 200 mesh-copper grids using a HT7700 TEM (Hitachi)^18^ operated at 80 kV with a Gatan numeric camera^19^. For the other samples, semi- (1 μm) and ultra-thin (100 nm) sections were sequentially cut from the same sample. Semi-thin sections were stained in 0.5% (w/v) TB solution for 15 min for correlative microscopy with TEM observations. Ultra-thin sections were handled on plastic rings for PATAg staining (Reis and Roland, 1974) and eventually transferred to 200 mesh-nickel grids. TEM observations were performed as described above. For quantitative analysis of DZ on PATAg images, wide views (×5K magnification) were chosen to provide the widest surface of analysis enabling to keep the ultrastructural details of PATAg-labeling, WT (*n* = 8) and *atltp2-1* (*n* = 8) hypocotyl epidermal cell cross-sections were carefully analyzed for quantification of DZ. The TEM images were individually computer-labeled using Corel Photopaint with red lines (detached cuticle) and green lines (outer periclinal cell wall). These color marks (length of red lines and length of green lines, both in μm) were measured with ImageJ on calibrated images. For each image, the ratio of detached cuticle length/total outer periclinal cell wall length (sum of red lines whenever present/green line) was calculated as %, and the results were expressed as mean % +/- standard error. Significant difference in *atltp2-1* as compared to WT was assessed by a Wilcoxon rank-sum test.

## Author Contributions

AJ and CP performed the experiments. YM and BP contributed to the histological analysis. FD performed the cuticular lipids analysis. EJ initiated the research. VB and VP conceived and designed the study, supervised the assays, analyzed and discussed the results. VP coordinated the writing of the manuscript. All authors participated to the writing and/or the drafting of the paper. All authors read and approved the final manuscript.

## Conflict of Interest Statement

The authors declare that the research was conducted in the absence of any commercial or financial relationships that could be construed as a potential conflict of interest.

## Footnotes

1 http://arabidopsis.info/

2 https://www.sigmaaldrich.com

3 http://www.hamamatsu.com

4 www.omegabiotek.com/

5 www.nanodrop.com

6 www.appliedbiosystems.com

7 www.lifescience.roche.com

8 www.thermofisher.com

9 www.microsoft.com

10 www.merckmillipore.com

11 http://www.riken.jp/en/

12 www.thermofisher.com

13 www.leica-microsystems.com

14 www.zeiss.fr/microscopy

15 https://imagej.nih.gov/ij/

16 www.fei.com/

17 www.emsdiasum.com/microscopy/

18 www.hitachi-hightech.com

19 www.gatan.com
