# Supplementary material for: The Arabidopsis Lipid Transfer Protein 2 (AtLTP2) Is Involved in Cuticle-Cell Wall Interface Integrity and in Etiolated Hypocotyl Permeability
Source: Front Plant Sci. 2017 Feb 27;8:263. doi: 10.3389/fpls.2017.00263 (PMC5326792; doi:10.3389/fpls.2017.00263)
Supplement: Supplementary file 1 [file Presentation_1.PDF]

Supplementary Material

The *Arabidopsis* Lipid Transfer Protein 2 (AtLTP2) is involved in cuticle-cell wall interface integrity and in etiolated hypocotyl permeability

Adélaïde Jacq[a], Clémentine Pernot[a, e], Yves Martinez[b], Frédéric Domergue[c], Bruno Payré[d], Elisabeth Jamet[a], Vincent Burlat[a] and Valérie Pacquit[a]

[a] Laboratoire de Recherche en Sciences Végétales, Université de Toulouse, CNRS, UPS, Castanet Tolosan, France
[b] Plateforme Imagerie-Microscopie, CNRS, Université de Toulouse, UPS, Fédération de Recherche FR3450 - Agrobiosciences, Interactions et Biodiversité, Castanet-Tolosan, France
[c] Laboratoire de Biogenèse Membranaire, UMR 5200 CNRS Université de Bordeaux, Bâtiment A3 - INRA Bordeaux Aquitaine, Villenave d'Ornon, France
[d] Centre de Microscopie Electronique Appliquée à la Biologie (CMEAB), Faculté de Médecine Rangueil, Toulouse III, Université P. Sabatier, Toulouse, France
[e] Present Address: University of Quebec in Abitibi-Témiscamingue, Rouyn-Noranda, Quebec, Canada J9X 5E4.

Correspondence:
Valérie Pacquit
pacquit@lrsv.ups-tlse.fr

Supplementary Figures and Tables

Supplementary Figure S1: Expression pattern of *AtLTP2* in WT, complemented lines and *atltp2-1*.

Supplementary Figure S2: Identification of AtLTP2 by MALDI-TOF MS analysis.

Supplementary Figure S3: AtLTP2-YFP is visualized only in plastids in transient expression experiments in *Nicotiana benthamiana* leaves.

Supplementary Figure S4: AtLTP2-TagRFP is retained in the modified ER in presence of Brefeldin A (BFA).

Supplementary Figure S5: Environmental scanning electron microscopy (ESEM) observations of etiolated hypocotyls along a developmental kinetic.

Supplementary Figure S6: Environmental scanning electron microscopy (ESEM) observations of 5-d-old etiolated hypocotyls from the various genetic lines.

Supplementary Figure S7: Quantitative analysis of detachment phenotypes observed by transmission electron microscopy (TEM).

Supplementary Table S1: Oligonucleotide primers.

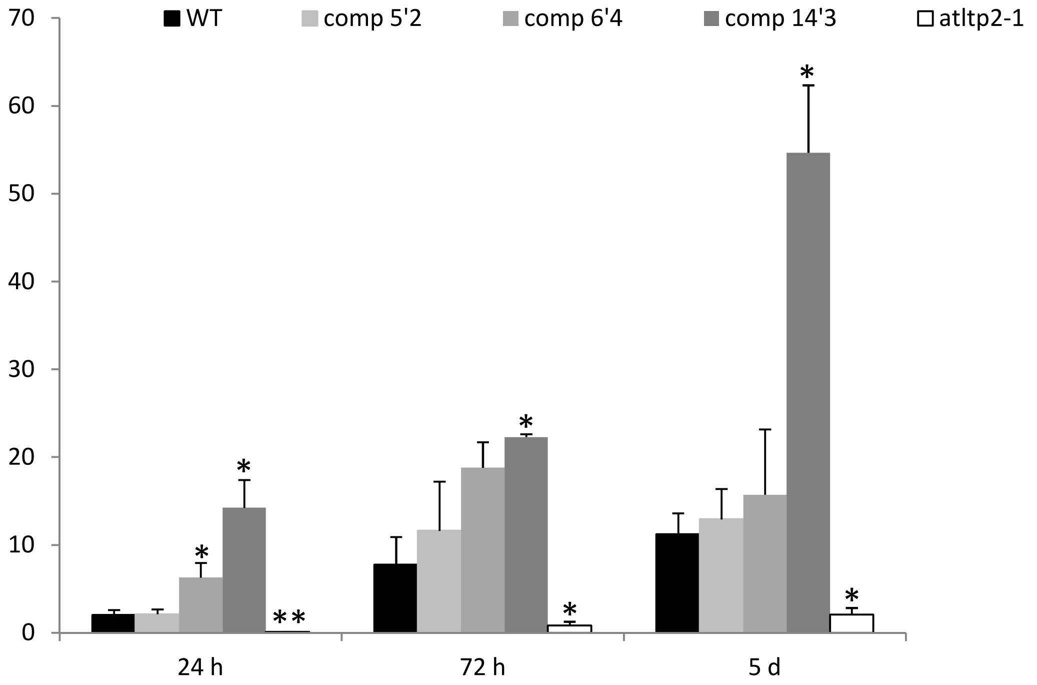

Supplementary Figure S1: Expression pattern of *AtLTP2* in WT, complemented lines and *atltp2-1*.

Relative *AtLTP2* gene expression was determined in 24-h-, 72-h and 5-day-old etiolated seedlings by RT-qPCR in WT (black), three complemented lines (shades of grey) and *atltp2-1* (white). Note that the expression level of *AtLTP2* is overall restored in complemented lines. Results represent the mean values ± SD (n=3). Significant differences from WT were assessed by a Student's t test (*, P<0.05; **, P<0.01).

(A)

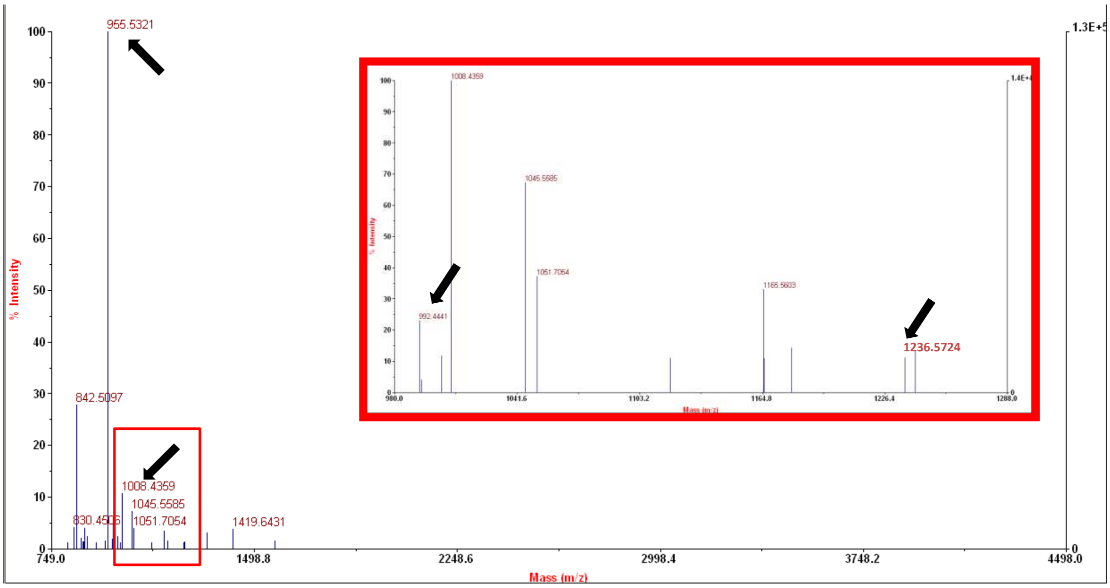

(B)

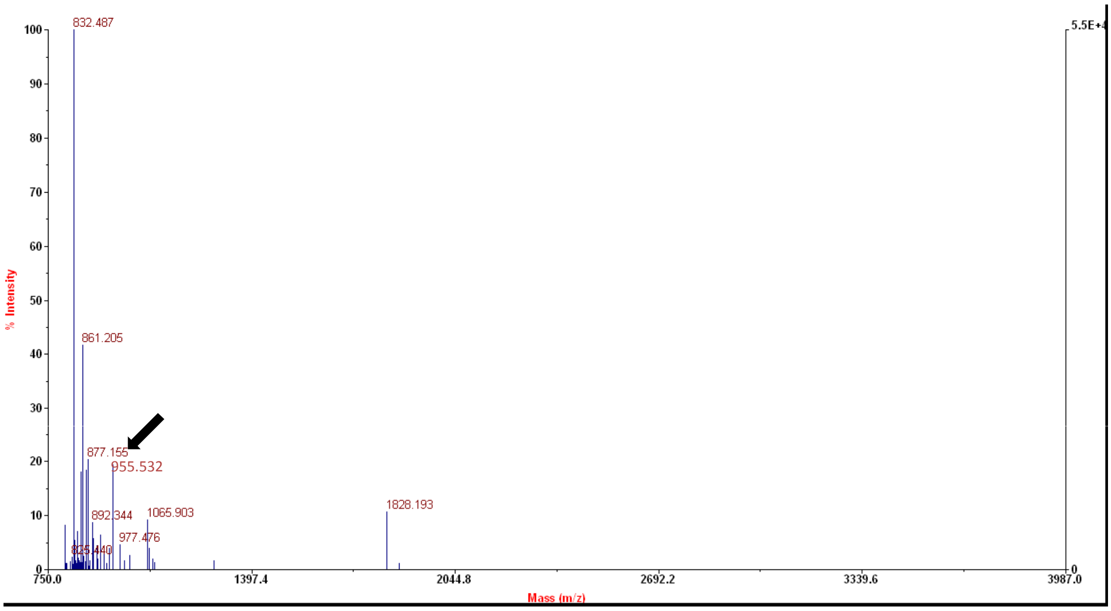

(C)

m/z Submitted	MH^+ Matched	Sequence
955.5321	955.5320	(K)AVGPGLNTAR(A)
992.4441	992.4466	(K)NMASTTPDR(Q)
1008.4359	1008.4415	(K)NM(oxidized)ASTTPDR(Q)
1236.5724	1236.6001	(K)ISASTNC(acrylamide modified)NTVR(-)

Supplementary Figure S2: Identification of AtLTP2 by MALDI-TOF MS analysis.

After 1D-E separation of proteins extracted from WT or *atltp2-1* etiolated hypocotyls stained bands at an apparent molecular mass of 9.6 kDa corresponding to that of AtLTP2 have been observed in WT and to a lesser extent in *atltp2-1* (see Fig. 1C). These two bands have been sampled. Proteins have been digested with trypsin and analyzed by MALDI-TOF MS. The spectra obtained with the WT and the *atltp2-1* samples are shown in panels **(A)** and **(B),** respectively. AtLTP2 has been identified thanks to four matching m/z found using ProteinProspector (prospector.ucsf.edu/prospector/mshome.htm) (panel C). The four peptides could be labeled on panel A and only one on panel B (black arrows). All of them were specific for AtLTP2 as checked using blastp on the Arabidopsis proteome at ncbi (blast.ncbi.nlm.nih.gov/Blast.cgi). The percentage of coverage of the sequence of the mature AtLTP2 was of 29 %.

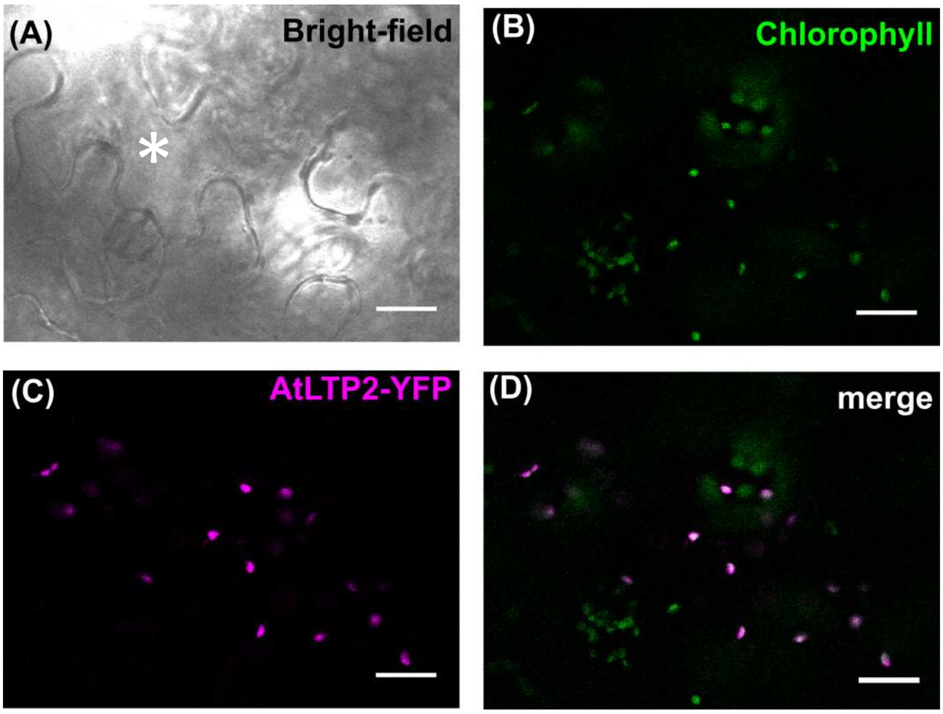

Supplementary Figure S3: AtLTP2-YFP is visualized only in plastids in *Nicotiana bentamiana* leaves.

AtLTP2 cDNA was first amplified with the primers (At2g38530-CDS-int-F and At2g38530-CDS-int-R, then Universal-attB1-primer and Universal-attB2-primer listed in Supplementary Table S1). It was cloned into the pDONR 207 vector and was fused in frame with *YFP* coding sequence into the pAM-PAT-35SS-GWY-YFP binary vector by using the Gateway® technology (Invitrogen™-ThermoFisher, www.thermofisher.com). After transformation with the recombinant binary vector, *A. tumefaciens* strain was infiltrated into *N. benthamiana* leaves. The bright-field **(A)** allowed the observation of cell morphology. The asterisk indicates the observed transformed cell. AtLTP2-YFP fluorescence was observed 48 h after agro-infiltration using confocal microscopy. AtLTP2-YFP fluorescence signal was false-colored in magenta **(C)**, chlorophyll auto-fluorescence was false-colored in green **(B)**. Merge appeared in white **(D)**. AtLTP2-YFP was observed in plastids but was not detected in the cell wall due to the instability of YFP (pKa 6.9) in this acidic compartment. Scale bars: 40 µm.

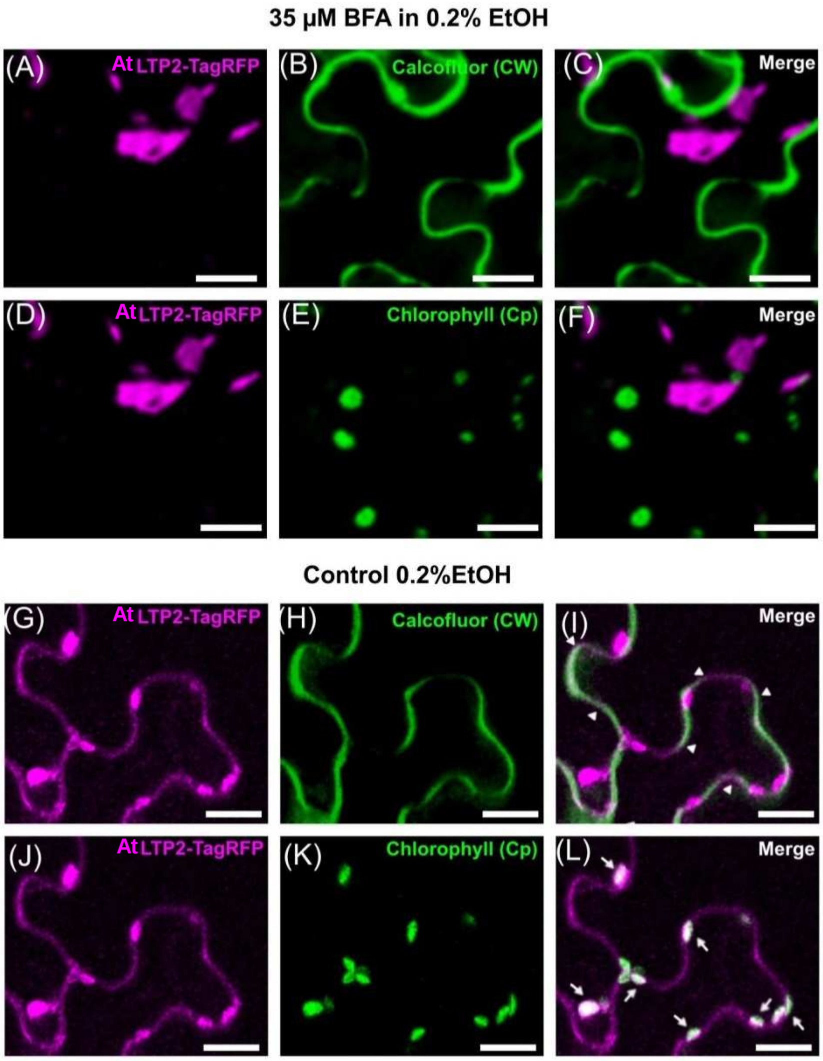

Supplementary Figure S4: AtLTP2-TagRFP is retained in the modified ER in presence of Brefeldin A (BFA).

Confocal images of AtLTP2-TagRFP localization were obtained 48 h after infiltration of *N. benthamiana* leaves. Transformed leaves were subjected to BFA treatment **(A-F)** or were mock-treated with 0.2% (v/v) ethanol (control 0.2% EtOH, **G-L**). AtLTP2-TagRFP fluorescence signal was false-colored in magenta **(A, D, G, J)**; cell wall calcofluor induced fluorescence (excitation: 365-395 nm; emission: 420 nm) **(B, H)** and chlorophyll auto-fluorescence **(E, K)** fluorescence were false-colored in green. Co-localized magenta and green fluorescence signals appeared in white in the merged images. Note that with the BFA treatment, AtLTP2-TagRFP fluorescence was excluded from calcofluor **(C)** and chlorophyll **(F)** fluorescence signals, **(I)**. Mock treatment shows the dual targeting of AtLTP2-TagRFP to the cell wall (**G-I**; arrowheads) and plastids (**J-L**; arrows). Legend: cell wall (CW); chloroplasts (Cp). Scale bars: 20 µm.

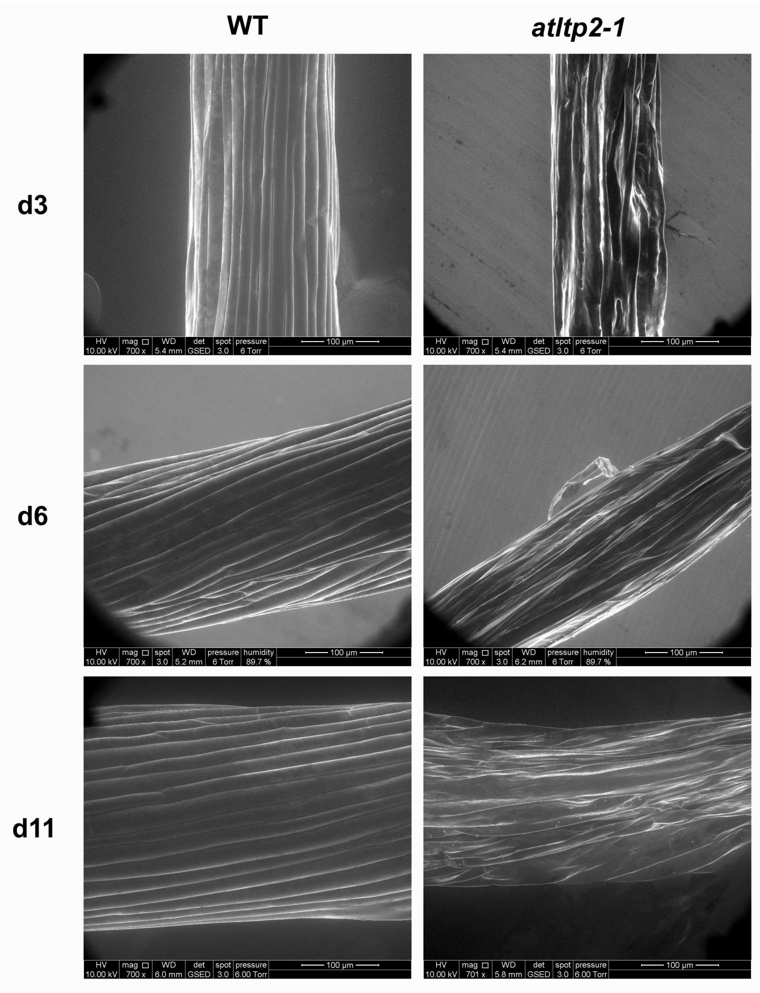

Supplementary Figure S5: Environmental scanning electron microscopy (ESEM) observations of etiolated hypocotyls along a developmental kinetic.

The WT (left column) and *atltp2-1* (right column) were compared at days 3, 6 and 11 as indicated on the left. Note the recurrent surface perturbation of *atltp2-1* as compared to the WT.

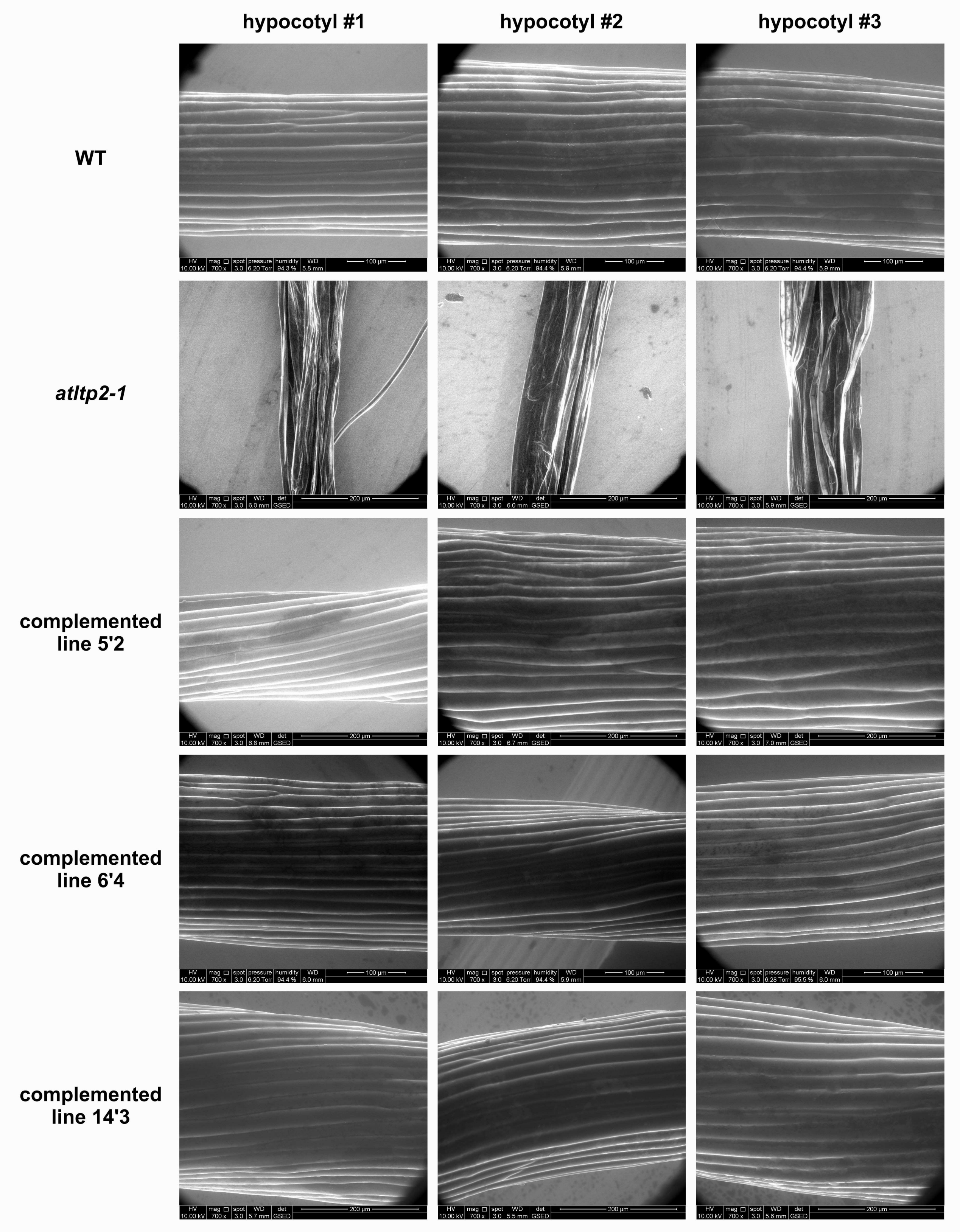

Supplementary Figure S6: Environmental scanning electron microscopy (ESEM) observations of 5-d-old etiolated hypocotyls from the various genetic lines.

Three individual 5-d-old etiolated hypocotyls from WT, *atltp2-1* and three selected complemented lines (labels on the left) were observed at x 700. Note the recurrent surface perturbation of *atltp2-1* as compared to WT and complemented lines.

Supplementary Figure S7: Quantitative analysis of detachment phenotypes observed by transmission electron microscopy (TEM). (See Figure next page).

Wide views (x 5K magnification was chosen since it provided the widest surface of analysis enabling to keep the ultrastructural details of PATAg-labeling) of WT (n= 8) and *atltp2-1* (n=8) hypocotyl epidermal cell cross-sections were carefully analysed for quantification of detachment zones. The TEM images were individually computer-labelled using Corel Photopaint with red lines (detached cuticle) and green lines (outer periclinal cell wall). The length of these color marks were measured with ImageJ on calibrated images. For each image, the ratio of detached cuticle length / total outer periclinal cell wall length (sum of red lines whenever present / green line) was calculated and expressed as %. A selection of views from WT and *atltp2-1* are displayed before and after computer analysis for comparison. The histogram shows the mean % +/- standard error (SE) of all the analyzed views for WT (n=8; total outer periclinal cell wall length 53.7 µm) and *atltp2-1* (n=8; total outer periclinal cell wall length 62.4 µm). Significant differences in *atltp2-1* as compared to WT was assessed by a Wilcoxon rank-sum test (***; p-value < 0,001). Note that the detachment zones between the cuticle and the cell wall were quantitatively (histogram) and qualitatively (more diffuse PATAg-reactivity) more pronounced in *atltp2-1* than in WT. All images are at the same scale indicated by bars.

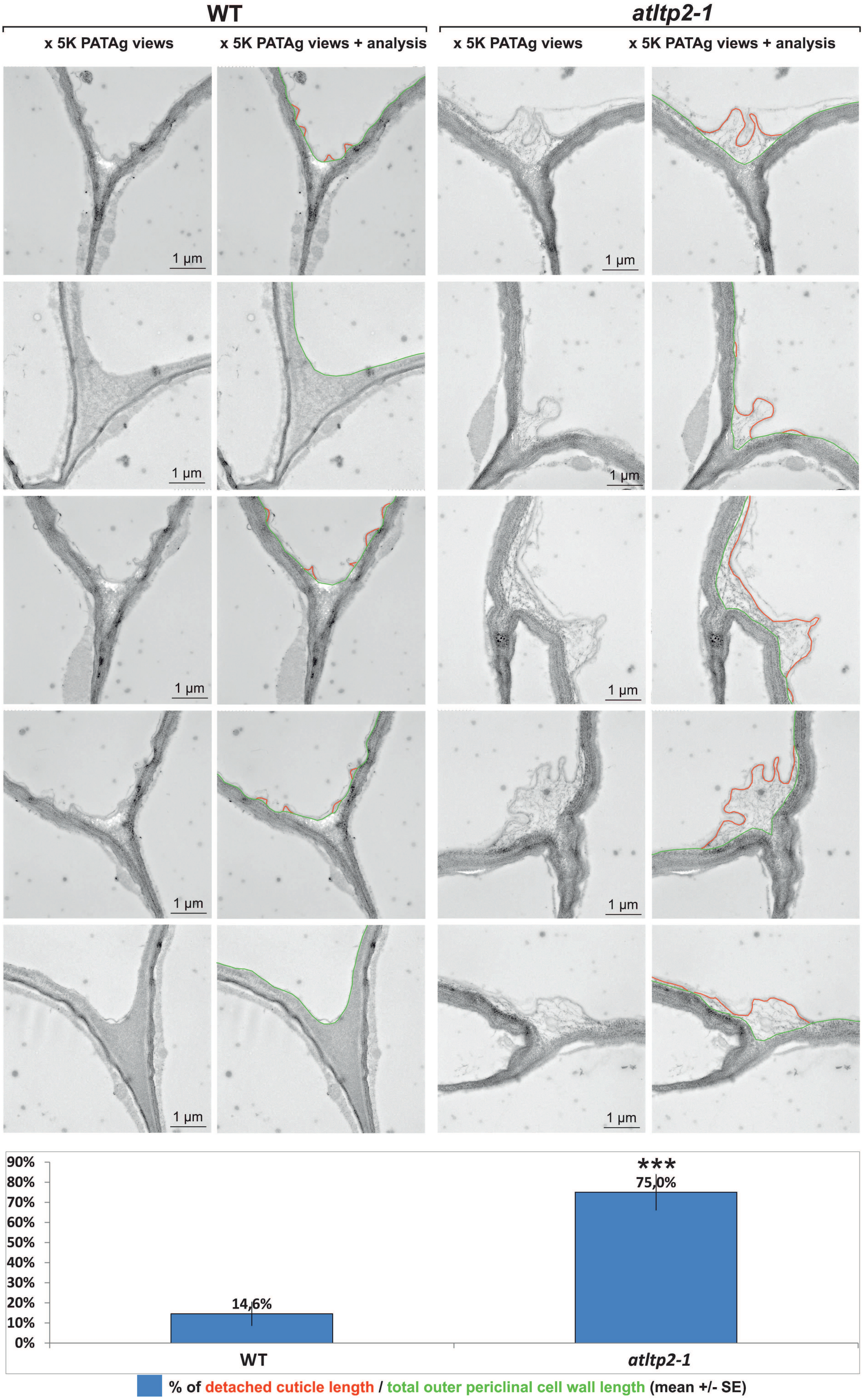
WT
atltp2-1
x 5K PATAg views
x 5K PATAg views + analysis
x 5K PATAg views
x 5K PATAg views + analysis
1 µm
90%
80%
70%
60%
50%
40%
30%
20%
10%
0%
***
75,0%
14,6%
WT
atltp2-1
% of detached cuticle length / total outer periclinal cell wall length (mean +/- SE)

Supplementary Table S1. Oligonucleotide primers

attB1-F-adapter	5’-ggggacaagtttgtacaaaaaagcaggct-3’	Gateway® cloning
attB2-R-adapter	5’-ggggaccactttgtacaagaaagctgggt-3’	Gateway® cloning
AttB2-TagRFP-stop-R	5’-agaaagctgggtcctaattaagtttgtgccccagtttgcta-3’	Sub-cellular localization of AtLTP2-TagRFP
Spacer-TagRFP-F	5’-tccggcggtggatccggcggaggtatggtgtctaagggcgaagagctga-3’	Sub-cellular localization of AtLTP2-TagRFP
AttB1-At2g38530-F	5’-aaaaagcaggcttcatggctggagtgatgaagttg-3’	Sub-cellular localization of AtLTP2-TagRFP
Spacer-At2g38530-R	5’-acctccgccggatccaccgccggacctcacggtgttgcagttg-3’	Sub-cellular localization of AtLTP2-TagRFP
At2g38530-CDS-int-F	5’- ggagatagaaccatggctggagtgatgaagttggca-3’	Sub-cellular localization of AtLTP2-YFP
At2g38530-CDS-int-R	5’-caagaaagctgggtccctcacggtgttgcagttggtg-3’	Sub-cellular localization of AtLTP2-YFP
Universal-attB1-primer	5’- ggggacaagtttgtacaaaaaagcaggcttcgaaggagatagaaccatg-3’	Sub-cellular localization of AtLTP2-YFP
Universal-attB2-primer	5’-ggggaccactttgtacaagaaagctgggtc-3’	Sub-cellular localization of AtLTP2-YFP
PromAtLTP2-F	5’-ttcggcccaaacccaacc-3’	Complementation of *atltp2.1*
TermAtLTP2-R	5’-cttacatttggaaagtcatgtttttcg-3’	Complementation of *atltp2.1*
PromAtLTP2-attB1-F	5’-aaaaagcaggctttcggcccaaacccaac-3’	Complementation of *atltp2.1*
TermAtLTP2-attB2-R	5’-agaaagctgggtcttacatttggaaagtcatgtttttcg-3’	Complementation of *atltp2.1*
At2g38530-Q-F	5’-tgccttcaatctgccgctaaag-3’	RT-qPCR
At2g38530-Q-R	5’-atttgaccgtcgctcatcacct-3’	RT-qPCR
At1g49240-F	5’-cacccgagaggaagtacagtg-3’	RT-qPCR
At1g49240-R	5’-catactctgccttagagatccaca-3’	RT-qPCR
At4g34270-F-qPCR	5’-gtgaaaactgttggagagaagcaa-3’	RT-qPCR
At4g34270-R-qPCR	5’-tcaactggataccctttcgca-3’	RT-qPCR
At5g15710-F-qPCR	5’-tttcggctgagaggttcgagt-3’	RT-qPCR
At5g15710-R-qPCR	5’-gattccaagacgtaaagcagatcaa-3’	RT-qPCR
LBa1-F	5’-tggttcacgtagtgggccatcg-3’	genotyping of *atltp2-1*
106-VB-For	5’-gacgaaccatctacatacaatctatgg-3’	genotyping of *atltp2-1*
106-VB-Rev	5’-gactttgcatgcgctaggaagtccag-3’	genotyping of *atltp2-1*
